# The design and development of a study protocol to investigate *Onchocerca volvulus*, *Loa loa* and *Mansonella perstans*-mediated modulation of the metabolic and immunological profile in lean and obese individuals in Cameroon

**DOI:** 10.1371/journal.pone.0285689

**Published:** 2023-06-02

**Authors:** Benjamin Lenz, Beng Amuam Andrew, Manuel Ritter, Indulekha Karunakaran, Narcisse Victor Tchamatchoua Gandjui, Lucy Cho Nchang, Jayagopi Surendar, Anita Obi Bate Ebob, Alexandra Ehrens, Ute Klarmann-Schulz, Arcangelo Ricchiuto, Janina M. Kuehlwein, Fanny Fri Fombad, Ambe Marius Ngwa, Tatiana Djikeussi Katcho, Achim Hoerauf, Samuel Wanji, Marc P. Hübner

**Affiliations:** 1 Institute for Medical Microbiology, Immunology and Parasitology, University Hospital Bonn, Bonn, Germany; 2 Parasite and Vector Biology Research Unit, Department of Microbiology and Parasitology, Faculty of Science, University of Buea, Buea, Cameroon; 3 Research Foundation in Tropical Diseases and the Environment, Buea, Cameroon; 4 German Center for Infection Research (DZIF), Bonn, Germany; 5 German-West African Centre for Global Health and Pandemic Prevention (G-WAC), Bonn, Germany; NIAID-ICER, INDIA

## Abstract

**Background:**

Life-style metabolic diseases are steadily rising, not only in developed countries, but also in low- and middle-income countries, presenting a global health problem. Metabolic disorders like type 2 diabetes and cardiovascular diseases are among the ten leading causes of death defined by the WHO in 2019. Results from animal and observational human studies suggest a connection between the decline in human helminth infections and rise of life-style-associated metabolic diseases in developing regions. This trial was designed to investigate filarial infections and their impact on metabolic diseases in Cameroon. We hypothesize that the induction of regulatory immune responses during filarial infection reduces obesity-induced low-grade inflammatory immune responses and thereby improves metabolic parameters, whereas anthelmintic treatment abolishes this protective effect.

**Methods/design:**

Participants infected with *Mansonella perstans*, *Onchocerca volvulus* and/or *Loa loa* being lean (BMI <25), overweight (BMI >25 and <30) or clinically obese (BMI ≥30) from Littoral regions of Cameroon will be evaluated for their parasitological, immunological, metabolic and biochemical profile before and after treatment of their parasitic infections. Anthropomorphic measurements and a detailed questionnaire will complement our analysis. The investigation will assess blood immune cell populations, serum adipokines and cytokines that could be influenced by the parasite infection and/or metabolic diseases. Further, parameters like blood glucose, homeostatic model assessment of insulin resistance (HOMA-IR), circulating lipids and circulating makers of liver function will be monitored. Parameters will be assessed before treatment, 12 and 18 months after treatment.

**Conclusion:**

The focus of this study is to obtain a comprehensive metabolic profile of the participants in rural areas of Cameroon and to investigate the relationship between filarial immunomodulation and metabolic diseases. This study will elucidate the effect of anti-filarial treatment on the metabolic and immunological parameters that partake in the development of insulin resistance, narrowing in on a potential protective effect of filarial infections on metabolic diseases.

**Trial registration:**

doi.org/10.1186/ISRCTN43845142, ISRCTN43845142 February 2020 Trial title Effects of filarial parasite infection on type 2 diabetes Issue date: 27.10.22, V.1.

## Background

Obesity and its associated metabolic diseases are a major public health problem on the global scale and an increasing challenge in low- and middle-income countries (LMIC) due to life-style alterations, cultural and social changes, ageing populations, increasing urbanization, dietary changes, reduced physical activity, unhealthy behavior and lack of awareness [1–5]. Obesity is a primary etiological factor for the development of type 2 diabetes (T2D), which accounts for approximately 90% of all diabetes cases [6, 7]. Obesity-induced insulin resistance is predominantly caused by continuous low-grade inflammatory processes, called meta-inflammation in multiple organs, which results from a combination of nutrient-energy stress and immune-metabolic dysfunction [8, 9]. In Africa, around 67% of diabetic subjects are undiagnosed and it is predicted that until 2045, the number of diabetes patients will increase by 134%. For instance, in Cameroon over half a million patients suffer from diabetes totaling in an age-adjusted prevalence of 5.5% according to the international diabetes federation (IDF) diabetes atlas in 2021.

Further, LMIC in sub-Saharan Africa display increased prevalence of neglected tropical diseases (NTDs), including helminth infections like filariasis. Human filariasis, an infection caused by parasitic filarial nematodes, can induce debilitating diseases. For example, onchocerciasis caused by *Onchocerca volvulus* can lead to severe dermatitis, visual impairment and ultimately result in vision loss.

An estimated 21 million were infected with onchocerciasis in 2017 leading to approximately 205 million disability-adjusted life years (DALY´s) [10]. In contrast, *Mansonella perstans*, with estimated 120 million people infected, is in general not associated with unambiguous clinical symptoms [11]. *Loa loa*, another filaria present in sub-Saharan Africa can cause pruritus, oedema, Calabar swelling and the transient migration of adult worms through the eye, giving it its common name African eye worm [12–17]. In order to escape protective host immune responses and to suppress the development of host pathology, filarial infections modulate the immune system of their hosts [18–27]. Parasitic filariae tilt the immune system towards a type 2 immune response, showing increased release of type 2 cytokines like IL-4 and IL-5, induction of eosinophilia and expansion of Th2 cells [18, 28, 29]. During chronic infection, filariae establish a regulatory milieu with increased levels of anti-inflammatory cytokines such as TGFβ and IL-10, as well as increased numbers of regulatory T cells [25, 30–34]. Because of these multifaceted interactions between parasitic helminths and its host, bystander immune responses can be impacted by filarial infections as well. Accordingly, autoimmune diseases like arthritis and type 1 diabetes [35–38], allergies and bacterial sepsis [39–42] showed a decrease in severity in mice infected with *Litomosoides sigmodontis*, a filarial parasite that induces similar immune responses as in human filarial infection [43–47]. Recent experimental studies in mice and cross-sectional reports in humans indicate potential connections between the reduction of human filarial and other helminth infections and the rise of autoimmune and life-style-derived metabolic diseases in LMIC. In case of diet-induced type 2 diabetes, prior research suggests that human filarial infections are protective [48]. Subsequent experiments in animal models of filariasis showed that infection of diet-induced obese mice with *L*. *sigmodontis* or *L*. *sigmodontis* extract treatment, improved glucose tolerance and restored the immune cell composition in adipose tissue, displaying increased numbers of eosinophils, innate lymphoid cells (ILC2) and AAM (alternatively activated macrophages) [49].

More recently, it was shown that treatment with a *L*. *sigmodontis* extract also increases adiponectin levels, suppressing the generation of pro-inflammatory Th1 and Th17 cells, which are associated with the development of diet-induced insulin resistance [50]. These murine experiments allowed the investigation of the causal relationship using knockout models, while human studies have until now only shown retrospective and cross-sectional associations between the absence of filarial infections and the increase of metabolic disease-associated parameters. For instance, the observational study in India showed that the prevalence of lymphatic filarial infections is lower in type 2 diabetes patients [48] in rural regions of China, a negative association between a history of *Schistosoma spp*. infections and glycemic parameters like glycated hemoglobin (HbA1C), fasting blood glucose and homeostatic model assessment of insulin resistance (HOMA-IR) was displayed [51]. Another study showed a negative correlation between diabetes prevalence and infection with an intestinal helminth *Strongyloides stercoralis* in Australian Aborigines [52]. Similarly, results from South India demonstrate that *S*. *stercoralis* infections are associated with an improvement of diabetes-associated and anti-inflammatory parameters that are in part reversed after anthelmintic treatment [53–60]. Further indications were provided by a survey in Flores Island, Indonesia, where individuals infected with soil-transmitted helminth infections displayed a reduction in HOMA-IR, indicating an improved insulin sensitivity [61, 62]. Finally, *Schistosoma haematobium* infected overweight/obese individuals in Lambaréné, Gabon, had improved circulating lipids (HDL). Following these surveys, and owing to a lack/scarcity of longitudinal data showing a causal association between helminth infections and the incidence of type 2 diabetes, this study was designed to (i) investigate the impact of filarial nematodes *O*. *volvulus*, *M*. *perstans* and *L*. *loa*, which have not been investigated so far for their immunomodulatory effect on the metabolic syndrome (dyslipidemia, insulin resistance and blood pressure hypertension) and type 2 diabetes and (ii) to assess the effect of anthelmintic intervention on the future development of insulin resistance and other metabolic complications.

These objectives will be accomplished by characterizing the metabolic and immunological profile of participants infected with *M*. *perstans*, *O*. *volvulus* and/or *L*. *loa* of lean, intermediate and obese BMI at the baseline and after comprehensive anthelmintic treatment and compare it to the profile of endemic, non-helminth-infected individuals. Potential changes in the host immune system and metabolic profile will be investigated by observing anthropomorphic parameters like weight, body mass index (BMI), waist circumference, body fat percentage and blood pressure. The primary objective of this study is the quantitative changes in insulin resistance (HOMA-IR) in lean, overweight and obese participants before and after treatment of the respective filarial infection. Secondary outcomes include changes in blood glucose, urea, liver enzymes, pancreatic hormones and gut hormones. In addition, measurement of a broad range of adipokines and cytokines as well as an analysis of the immune cell composition and phenotyping using flow cytometry will be used to depict a detailed profile of the participant’s immune and metabolic profile before and after anthelminthic intervention.

## Methodology/design

### Overall study design

**Fig 1:**
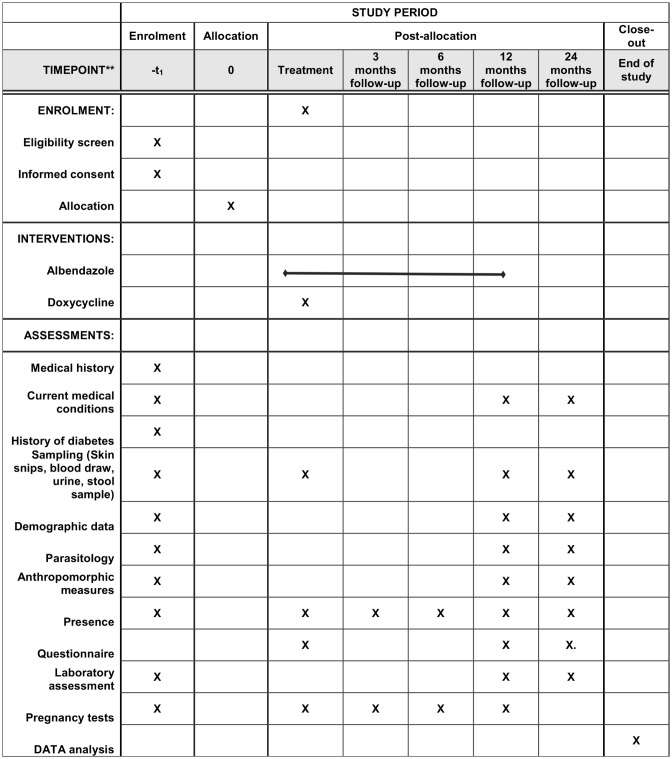
Participants’ timeline.

This study was designed as a partially controlled, open label pilot trial to investigate the impact of filarial infections on the metabolic and immunological profile before and after anti-filarial therapy in lean, overweight and obese participants. The participants’ timeline is depicted in Fig 1.

The major aim is to elucidate a potential protective role of chronic filarial infections with *M*. *perstans*, *O*. *volvulus* and/or *L*. *loa* on the onset and progression of metabolic diseases like type 2 diabetes (T2D) by improving metabolic parameters such as HOMA-IR and chronic low-grade inflammation in all participants. A comprehensive evaluation of changes in metabolic markers like liver enzymes, gut and pancreatic hormones, blood glucose and pro- and anti-inflammatory markers, e.g. the adipokines adiponectin, leptin and visfatin as well as cytokines will be performed. Further, the immediate state and response to stimuli of the participant’s immune system will be determined through *in vitro* cultures and flow cytometry in all filarial-infected and uninfected patients. The second objective of the study is to identify changes in the metabolic and immunological profile in patients after treatment of the filarial infection and if this leads to a metabolic and immunological profile similar to uninfected participants or whether observed changes remain altered despite filarial treatment. Finally, platelet count, absolute white blood cell counts for eosinophils, neutrophils, basophils, monocytes and lymphocytes as well as HbA1c and hemoglobin will be monitored for each participant throughout the whole study.

### Trial setting

Participants that consented to partake in the study (age 18–65) will be recruited from several scouted sites in the rural areas around the Littoral region of Cameroon. Mapping of the filarial infections showed an incidence of the respective parasites *O*. *volvulus* (4.9–21.9%), *L*. *loa* (1.4–22.5%) and *M*. *perstans* (0.2–16.9%) (Table 1) within the study regions. The rural communities are close to the field medical research station in Manjo to ensure a quick transfer of the samples from the field to the site of sample storage and analysis. Half of the samples will be stored in Cameroon and the other half will be stored in Bonn, Germany. Storage and use of theses samples will be specifically addressed to the participants in combination with the informed consent form (S1 File and S1 Checklist).

**Table 1:** Prevalence of *O*. *volvulus*, *M*. *perstans* and *Loa loa* infections in the study area.

Health district	*M*. *perstans* prevalence	*O*. *volvulus* prevalence	*L*. *loa* prevalence	Screened persons (total)
Yabassi	16.9%	21.9%	22.5%	1225
Manjo	0.2%	4.9%	1.4%	2429
Loum	13.9%	17.3%	18.2%	583
Melong	1.4%	4.9%	2.9%	1748

### Trial governance

Ethics approval to perform this study was obtained from the Cameroon National Ethics Committee for Human Health Research (No 2019/03/CE/CNERSH/SP). In Germany, ethical approval was obtained by the Ethic Committee for Clinical Human Research (Nr. 046/18). Protocol amendment will be re-submitted to the ethics boards for approval. The clinical trial was registered under ISRCTN43845142 (doi.org/10.1186/ISRCTN43845142). Written informed consent will be obtained from respondents after ensuring that the participant understood and accepted their role in the study.

### Study population

**Fig 2:**
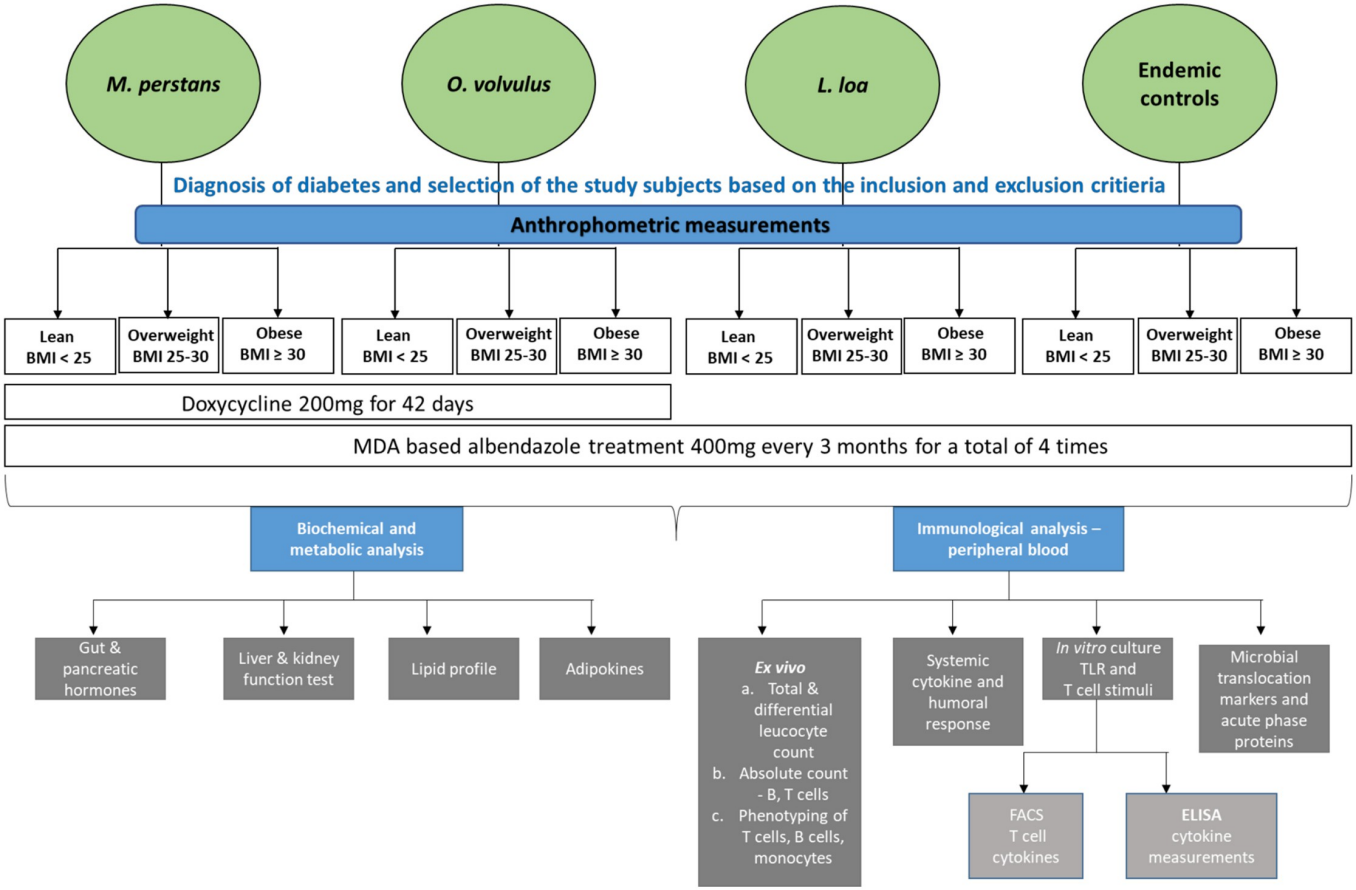
Overview of the study. *M*. *perstans*, *O*. *volvulus-* and/or *Loa loa*-infected participants and non-helminth-infected endemic controls will be enrolled in this study and grouped according to their BMI as lean (BMI<25), overweight (BMI 25–29) or obese individuals (BMI≥30). *M*. *perstans* and *O*. *volvulus* positive participants will receive 200 mg doxycycline for 42 days. All participants will receive 400 mg of albendazole every three months to treat intestinal helminth infections. Before, 12 month and 18 months after doxycycline treatment, anthropomorphic, metabolic and immunological measurements will be performed as indicated in the figure.

The first phase comprises a cross sectional study containing four main study groups (Fig 2). All participants will be evaluated according to the inclusion and exclusion criteria listed in Table 2. Group 1 consists of *M*. *perstans*-infected participants who tested positive for microfilariae in the prick blood smear and/or the *M*. *perstans*-specific loop-mediated isothermal amplification (LAMP) [63]. Group 2 will include *O*. *volvulus*-infected individuals that show at least one palpable onchocercoma and are tested positive in a diagnostic Ov150 (*Onchocerca volvulus* antigen 150) LAMP [64] or PCR and/or are skin snip positive for microfilariae. Group 3 consists of endemic normal individuals that have lived in the endemic study area for at least five years, have normal eosinophil frequencies (<4%), and are negative for microfilariae by microscopy and PCR. Group 4 includes *L*. *loa*-infected participants that have detectable microfilariae by microscopy or LAMP. Co-infections with mentioned filariae are included in group 1, 2 and 4. Stool samples from each participant will also be tested for intestinal helminth infections. Presence of intestinal helminths in kato-katz is no exclusion criteria for groups 1, 2 and 4. Upon recruitment, anthropomorphic measurements and the immunological and metabolic profile of each participant will be analyzed. All participants will be asked to be in an overnight fasting state before the clinical parameters are taken. The importance of fasting before the trial will be communicated during the sensitization of the health districts. The next phase of the study will be an intervention study that focuses on analyzing the changes in the metabolic and immunological profiles after anthelmintic treatment. The goal is to provide direct evidence of the filariae-mediated protective effect and to investigate the causal relationship between absence (loss) of filarial infection and a disadvantageous metabolic profile and increased inflammation. In this regard, all *M*. *perstans-* as well as *O*. *volvulus*-infected lean, overweight and obese participants (including *L*. *loa* co-infected individuals) will be recruited in the cross-sectional study for anti-filarial therapy with 200 mg doxycycline for 42 days (for individuals with >50 kg body weight; individuals with 40–50 kg body weight will be treated with 100 mg doxycycline for 42 days). Participants from all groups will be treated with a single dose of 400 mg albendazole every three months with four treatments in total to eliminate all intestinal helminth infections. All participants will be followed up at 12 and 18 months after treatment and a selection of immunological and metabolic parameters that were determined in the first part of this study will be analyzed again. Patients will be screened for filarial and intestinal helminth infections and the impact of possible re-infections following albendazole and doxycycline treatment will be analyzed. Compliance to the treatment will be confirmed by collecting empty drug containers during the visit of the study subjects. Subjects will be monitored throughout the study for any adverse effects.

**Table 2:** Inclusion and exclusion criteria.

**Inclusion criteria**	Males and females between 18–60 years old Body weight > 40kg Resident in the endemic area for at least 5 years Good general health without any clinical condition requiring long-term medication Willingness to participate in the study as evidenced by signing the Informed Consent Form Negative pregnancy test
**Inclusion in *O*. *volvulus* positive**	*O*. *volvulus* patients with at least one palpable onchocercoma, microfilariae skin snip positive and/or LAMP/PCR positive for *O*. *volvulus*
**Inclusion in *M*. *perstans* positive**	*M*. *perstans* patients positive for microfilariae and/or LAMP/PCR
**Inclusion in *L*. *loa* positive**	*L*. *loa* patients positive for microfilariae and/or LAMP/PCR
**Inclusion in endemic normal**	Endemic controls, judged by absence of microfilariae, palpable onchocercoma, LAMP/PCR negative for *M*. *perstans*, *O*. *volvulus* and *L*. *loa*. Possess normal eosinophil frequencies (0.5–4%).
**Exclusion criteria**	Positive pregnancy test Lactating mothers Last intake of ivermectin (IVM) less than 4 months ago Intake of anti-filarial antibiotics (tetracycline) less than 12 months ago Evidence of tuberculosis (clinical aspects) Evidence of clinical aspects of HIV infection Evidence/previous diagnosis of chronic diseases (urolithiasis, liver cirrhosis, congestive heart failure, chronic lung diseases, chronic infections other than filariae, viral hepatitis) Evidence of autoimmune diseases and allergies Evidence of acute infection (haematuria, cough, fever). Evidence of clinically significant neurological, cardiac, pulmonary, metabolic, malaria, rheumatologic or renal disease as far as can be assessed by history of individuals, physical examination, and/or laboratory examinations Childbearing potential and not willing or able to use methods to prevent a pregnancy for the entire treatment duration in addition to hormonal contraception (e.g. condoms) unless surgically sterilized/ hysterectomized or any other criteria considered sufficiently reliable by the investigator Behavioural, cognitive or psychiatric disease that in the opinion of the trial clinician affects the ability of the participant to understand and cooperate with the study protocol
**Laboratory exclusion values**	Haemoglobin < 8 g/dL Neutrophil count < 500/μl Platelet count < 100 000/μl Creatinine > 2 times upper limit of normal AST (GOT) > 2 times upper limit of normal ALT (GPT) > 2 times upper limit of normal γ-GT > 2 times upper limit of normal HbA1c above 44 mmol/mol Hb (6%) Abnormal white blood cell counts below or above (3,5–11,3 x 10^3^/μl)

### Referral system

All newly diagnosed diabetic subjects will not be included in the study and will be referred to a local public health center or to a private practitioner specialized in diabetes care, whose details will be provided by field investigators on request. Anthropometric measurements, blood pressure readings, and capillary glucose values will be immediately conveyed to study subjects and instructions to seek medical attention or adopt life-style measures will be provided when results deviate from the normal range. Biochemical parameters and electrocardiogram (ECG) results and if needed instructions to seek medical attention, will be mailed to the subjects later.

### Definitions/Evaluation criteria for metabolic abnormalities

#### Diabetes

Diabetes will be defined by prior diagnosis and current use of medications for diabetes (insulin or oral hypoglycemic agents) as well as/or fulfillment of criteria laid down by the WHO-IDF Consultation Group Report (2006) [65], i.e., fasting capillary blood glucose ≥126 mg/dl or 2 h capillary post-glucose value ≥200 mg/dl. Hemoglobin A_1C_ ≥6.5% will be considered as diabetes according to the American Diabetes Association criteria (2014) guidelines [66].

#### Insulin resistance

Insulin resistance will be calculated using formula for the HOMA-IR: fasting insulin (μIU/ml) × fasting plasma glucose (mmol/l)/22.5 [67].

#### BP/ hypertension

Hypertension will be diagnosed based on past medical history and drug treatment and/or if the subjects had systolic blood pressure (SBP) of 140 mmHg or greater and/or diastolic blood pressure (DBP) of 90 mmHg [65].

#### General obesity

BMI will be calculated as weight/(height)2[kg/m^2^]. Over-weight will be defined as BMI ≥25.0–29.9 kg/m^2^ and obesity as BMI ≥ 30.0 kg/m^2^.

#### Abdominal obesity

Abdominal/central obesity will be defined as waist circumference (WC) ≥ 90 cm in men and ≥80 cm in women according to the IDF criteria (2006) [65].

#### Dyslipidemia

National Cholesterol Education Programme (NCEP-ATP) guidelines will be used for definitions of dyslipidemia [68].

- Hypercholesterolemia: Serum cholesterol levels ≥200 mg/dl (≥5.2 mmol/liter) or drug treatment for hypercholesterolemia
- Hypertriglyceridemia: Serum triglyceride levels ≥150 mg/dl (≥1.7 mmol/liter) or drug treatment for hypertriglyceridemia
- Low High-Density Lipoprotein (LDL) cholesterol: High-density lipoprotein cholesterol levels <40 mg/dl (<1.04 mmol/liter) for men and <50 mg/dl (<1.3 mmol/liter) for women
- High Low-Density Lipoprotein (HDL) cholesterol: Low-density lipoprotein cholesterol levels ≥130 mg/dl

### Metabolic syndrome

Metabolic syndrome will be diagnosed according to the IDF criteria (2006) [65]. For a person to be defined as having the metabolic syndrome they must have central obesity (defined as WC ≥90 cm for males and ≥80 cm for females) and any two of the following four factors:

- Triglycerides ≥150 mg/dL (1.7 mmol/L) or specific treatment for this lipid abnormality
- HDL cholesterol <40 mg/dL (1.03 mmol/L) in males <50 mg/dL (1.29 mmol/L) in females or specific treatment for this lipid abnormality
- Blood pressure systolic BP ≥130 or diastolic BP ≥85 mm Hg or treatment of previously diagnosed hypertension
- Fasting plasma glucose (FPG) ≥100 mg/dL (5.6 mmol/L) or previously diagnosed type 2 diabetes.

## Evaluation criteria and methods

### Questionnaire and anthropometric measurements

A structured questionnaire will be used to obtain data on demographic and socioeconomic parameters like education, religion, family status, occupation, family history of metabolic diseases and income. In addition, the participants will be asked to describe their dietary habits, extent of daily physical activities and sport in depth to closely observe and evaluate the living conditions.

The diet questionnaire is designed to gather information on the carbohydrate, fat and protein intake, fruit and vegetable consumption as well as the number of sugared drinks and candy. Further, the complete medical history and family history of diabetes and cardio-vascular disease will be gathered. Anthropometric measurements will complement the questionnaire. The participants’ age, gender, hip and waist circumference measures will be taken. The weight, body fat, muscle mass, visceral fat and calorie intake will be measured using a body analyzer (Body analyzer HBF-511B-E Omron, Mannheim, Germany). Blood pressure will be recorded from the right arm of each participant. In addition, platelet count and density, white blood cell counts for eosinophils, neutrophils, basophils, monocytes and lymphocytes as well as HbA1c and hemoglobin will be assessed for each participant. Capillary blood glucose will be assessed using Accu-Check-Aviva glucometer and test strips (Roche, Basel, Switzerland). Finally, 12-channel resting ECG (IG ECG series 12 Channel ECG machine, International Group medical technology and electronics GmbH, Bremen, Germany) will be performed.

### Intestinal helminth infections

Intestinal helminth infections and additional protozoa will be detected using Kato-Katz from stool samples. In detail, a single fresh stool samples will be taken from each patient at before treatment, 12 and 18 months after treatment. A small amount of stool sample will be placed on a newspaper and covered with a small piece of nylon. Using a spatula, the sample will be pressed through the nylon. The sieved stool will be distributed evenly using a calibrated template for egg quantification. A methylene blue glycerol-soaked cellophane piece will be placed over the stool sample. Finally, a glass slide will be placed on top of the stool sample and pressed down until the writing of the newspaper can be seen through. The glass slide will be removed carefully and placed under a microscope.

### Detection of filarial infections

*O*. *volvulus* infections will be assessed via skin biopsies of 2 mm diameter at the posterior iliac crest, which will be placed in a 96-well plate containing PBS. Microfilariae contained in the skin snip leaving the biopsy tissue will be counted.

Skin biopsies from the left and right side will be taken for each participant. *M*. *perstans*, *L*. *loa* and other filarial infections will be detected via blood prick test followed by staining and microscopy. Due to the absence of *Wuchereria bancrofti* at the study site, a collection of blood at night is not necessary. A LAMP of the skin snips and whole blood will be used as a validation for skin and blood dwelling filarial infections as an additional assay to determine the possible co-infection [69, 70].

### Determination of the metabolic and immunological profile

Serum will be obtained from blood drawn into 5 ml serum clot activator tubes (BD Vacutainer^®^, Franklin lakes, NJ, USA), incubated for 20 min and centrifuged at 2000 g for 10 min (Human Biochemica und Diagnostica GmbH, Wiesbaden, Germany). The serum phase will be aspirated using 2 ml serological pipettes and analyzed directly at the study center while the rest will be aliquoted into cryopreservation tube (Greiner Bio-One, Solingen, Germany). Cryopreservation tubes (VWR International, Radnor, PA, USA) will be frozen and transported at -20°C. The metabolic state of the participants will be captured in depth by analyzing the circulating lipid and lipoprotein composition, the circulating liver-enzymes, parameters of the kidney function, as well as markers for the overall metabolic equilibrium. Participant’s serum and urine will be collected at the study site and analyzed at the study coordinating center using a HumaStar200 auto-analyzer (Human Biochemica und Diagnostica GmbH, Wiesbaden, Germany). The lipid profile (cholesterol, triglyceride, HDL and LDL), liver enzymes (AST, alanine-aminotransferase (ALT), alkaline phosphatase (ALP), y-glutamyltransferase (y-GT)), kidney function (creatine, urine urea and fasting albumin), C-reactive protein and glucose will be analyzed using the HumaStar200. Further serum analysis will be performed using custom designed Luminex ProcartaPlex assays (Thermo Fisher Scientific, Waltham, Massachusetts, USA) to investigate the cytokine and chemokine profile and metabolic status between groups and time points, e.g. upon study inclusion, 12 and 18 months after the treatment. The focus will be an analysis and comparison of a broad range of chemokines and cytokines: Pro- and anti-inflammatory cytokines (IL-1β, IL-2, IL-5, IL-13, IFNγ, IL-9, IL-17) as well as eosinophil specific parameters like Eotaxin 1/CCL-11 and Eotaxin 2/CCL-24, and RANTES/CCL-5.

The following metabolic parameters will be assessed with ProcartaPlex (Thermo Fisher Scientific, Waltham, Massachusetts, USA): adiponectin, leptin, visfatin, resistin, plasminogen activator factor (PAI-1), pancreatic hormones (glucagon, insulin, C-peptide), gut hormones (ghrelin, gastric inhibitory polypeptide (GIP), glucagon like peptide 1 (GLP-1)) and acute-phase proteins (SAA). Multiplex assays will be performed according to the manufacturer’s protocol. Cytokines and chemokines will be additionally measured in the supernatant upon whole blood stimulation. Therefore, all samples will be stimulated with anti-CD28/CD49 antibodies (BioLegend^®^, San Diego, CA, USA, Ultra-LEAF^™^ purified anti-human antibody), as well as the corresponding stimuli Pam3Cys (100 ng/ml Roche, Basel, Switzerland), lipopolysaccharide from *Salmonella minnesota* (100 ng/ml, Merck, Darmstadt, Germany) or *Onchocerca ochengi* antigen (provided by Prof. Wanji, Buea, Cameroon) with and without palmitic acid (Sigma-Aldrich, ST. Louis, MI, USA) for 18 h at 37°C and 5% CO_2_. Cells will be separated from the supernatant by centrifugation at 1500 g for 5 min. The supernatant will be carefully removed and transferred into microdilution tubes (STARLAB International GmbH, Hamburg, Germany) and stored at -20°C. The ELISA and/or Luminex assays will be performed according to the manufacturer’s protocol.

### Immune cell phenotyping by intracellular cytometry

Whole blood from 50 randomly selected patients of each group will be collected in 11 ml BD Vacutainer^™^ sodium heparin blood-collection tubes. Whole blood (100 μl) will be plated in 96-well round bottom wells (Cellstar^®^, Greiner Bio-One, Solingen, Germany) for each condition. All samples will be stimulated with anti-CD28/CD49 antibodies (Ultra-LEAF^™^ Purified anti-human Antibody; BioLegend^®^, San Diego, CA, USA) as well as phytohaemagglutinin (10 μg/ml; Sigmar-Aldrich, ST. Louis, MI, USA) or *O*. *ochengi* antigen with and without palmitic acid (Sigma-Aldrich). Cells will be stimulated for 2 hours before addition of brefeldin A (Sigma-Aldrich) and the incubation will be continued for an additional 16 hours. After stimulation, cells will be centrifuged and red blood cells (RBC) lysed twice by incubation with RBC-lysis buffer (Roche, Basel, Switzerland).

Finally, cells will be washed and fixed using fixation buffer (Biolegend^®^, San Diego, CA, USA). The fixed cells will be stored at -20°C in freezing media containing RPMI1640 (Thermo Fisher Scientific, Waltham, Massachusetts, USA) with 10% dimethyl sulfoxide (Carl Roth, Karlsruhe, Germany) and 20% fetal calf serum (Pan-biotech, Aidenbach, Germany). Frequencies and activation of naïve, central memory and effector memory T cells, Th1, Th2, and regulatory T cells as well as naïve, memory, immature and plasma B cells and monocytes will be analyzed from the cryopreserved cell solution using flow cytometry.

### White blood cell analysis

The peripheral blood cell composition of all patients will be investigated using the Horiba Yumizen H500 white blood cell analyzer (Horiba Europe GmbH, Wiesbaden, Germany). The device will be used to measure frequencies and numbers of eosinophils, neutrophils, basophils and lymphocytes. Furthermore, platelet count and volume as well as red blood cell count and volume will be analyzed.

### Intervention protocol

**Doxycycline:** Individuals infected with *M*. *perstans* and *Onchocerca volvulus* will be treated with a daily dose of 200 mg doxycycline for six weeks. Individuals with less than 50 kg body weight will receive 100 mg doxycycline for six weeks. Pregnancy tests will be done before starting the treatment and every two weeks during the doxycycline treatment to prevent doxycycline treatment in pregnant women. Participating women will be advised to use contraception measures and willingness to comply is an inclusion criterion. In case of pregnancy during doxycycline treatment, treatment will be immediately stopped and women will be referred to a local clinic and monitored until the end of pregnancy for potential side effects of prior doxycycline treatment.

**Albendazole:** All participants in this study will be treated with albendazole (before the first doxycycline treatment in the respective group) to remove intestinal helminths and to complement anti-filarial therapies. Therefore, individuals will receive a single dose of 400 mg albendazole every three months with four treatments total.

### Treatment efficacy

The treatment efficacy at 12 months and a potential reinfection will be monitored at 18 months after treatment using skin snips, diagnostic PCR, thick blood smears and Kato-Katz.

### Choice of comparator

Doxycycline is a macrofilaricidal drug for *Wolbachia*-containing filariae such as *O*. *volvulus* and *M*. *perstans*. Ivermectin, which is used for MDA, has no prominent macrofilaricidal efficacy. Albendazole, is the standard drug used for intestinal helminths. Therefore, doxycycline and albendazole are justified as comparator.

### Sample size

This is a pilot study to exploit for the first time the impact of infections with the filarial nematodes *L*. *loa*, *O*. *volvulus* and *M*. *perstans* on the metabolic profile and the systemic immune response in lean, overweight and obese patients. As there is limited data to perform a thorough sample size calculation, we will use 200 persons per group for this pilot trial (*O*. *volvulus* infected, *M*. *perstans* infected, *L*. *loa* infected and endemic non-helminth-infected persons). Thus, individuals with active filarial infections and helminth-free individuals will be included at a case: control ratio of 1:1:1:1. Cameroon has an estimated age-adjusted diabetes prevalence of 5.5% according to the international diabetes federation (IDF) diabetes atlas in 2021. The prevalence of *O*. *volvulus*, *L*. *loa* and *M*. *perstans* in the rural study areas are between 4.9–21.9%, 1.4–22.5% and 0.2–16.9%, respectively (Table 1). Based on previous trials conducted by the team of Prof. Wanji in Cameroon, we expect drop-out rates of around 20%.

### Recruitment

Potential participants will be familiarized with the study by social scientists before screening start. The sensitization will include study advertisement and clear depiction of the participation benefits and risks as well as the location of the study centers. A comprehensive pre-screening of the study sites will be performed. 6000 participants will be screened for their infection status to ensure a sufficient number of filariasis patients. Screening will be stopped if I) 6000 potential participants were screened or II) the group allocation is completed beforehand. Participants will be allocated into their respective groups using the measured BMI and infection status. Sensitization, screening, enrollment and follow-up will be performed by a highly experienced field team from Prof. Wanji, which includes social scientists, parasitologists, nurses, medical doctors and drivers.

### Retention plan

Participant retention is promoted by providing albendazole treatments every three months and connecting the 12 months albendazole treatment with the 12-months follow-up and therefore giving increased incentive to participate. In addition, the free medical examination, ECG, white blood cell counts, overnight fasting blood glucose and biochemistry test might provide an additional incentive. Further, a free meal is provided for all attending participants, due to the overnight fasting requirement. Potential outcomes for the participants are listed in the Table 3.

**Table 3:** Trial variables and predicted outcomes.

Variable/Outcome	Hypothesis	Parameters measured
Primary Outcome	*O*. *volvulus*, *M*. *perstans* and *L*. *loa* infection improve HOMA-IR Clearance of the adult filariae (*O*. *volvulus*, *M*. *perstans*) by doxycycline treatment abolishes the protective effects of *O*. *volvulus* and *M*. *perstans* infection	HOMA-IR (blood glucose, insulin)
Secondary Outcome	Infection with *O*. *volvulus*, *L*. *loa* or *M*. *perstans* reduce risk factors to develop type 2 diabetes Type 2 diabetes is negatively associated with filarial infection Co-infections have additional effects on the metabolic and immunological parameters There is an adverse impact on the metabolic and immunological profile 12 and 18 months after treatment of filarial infection Reinfection changes the metabolic and immunological profile	Microfilariae counts, HbA1c, waist circumferences, fasting blood glucose, LDL, HDL, ALP, AST, yGT, GOP, GTP, CRP, triglyceride, cholesterol, creatinine, adipokines and cytokines, treatment success, white blood cells, blood pressure, egg count in stool, risk factors according to questionnaire
Sub-Group Analysis	Sex and age, food intake, living condition, income, physical activity significantly impact the metabolic profile	Demographic data, participant data obtained via the questionnaire

### Data management and statistical analysis

High level of confidentiality will be ensured to safeguard the personal information of the study participants and other data and such information will be made accessible only to authorized personnel and the study’s principal investigators. All data collected in the paper CRF will be stored under strict confidentiality and entered electronically in the data collection program REDCap ((https://www.project-redcap.org/) REDCap Consortium Emory University, Atlanta, USA) [71, 72] and verified by double data entry. The paper CRF will be stored in locked rooms in the central laboratory at the study site. All statistical analyses will be performed using SPSS version 16.0 software. Hard copies like questionnaires and ECG results will be stored for 15 years and password-protected electronic databases for 35 years. Missing data will be excluded from the primary evaluation. A second analysis will be performed using multiple imputation to evaluate the potential impact of the missing data. The study’s principal investigators as well as the data management team will be able to access the collected trial data.

### Primary outcome assessment

The primary outcome of this study is the quantitative change in insulin resistance (HOMA-IR) in lean, overweight and obese participants before and after treatment of their respective filarial infection. Comparisons will be made using two-sided hypotheses with α = 0.05 under the assumption that filarial infection improves insulin resistance and treatment of filarial infections will reduce their protective effect and increase the risk to develop insulin resistance. The statistical test used is the two-sided Fisher’s Exact Test or Chi-squared test. Additionally, 95% confidence intervals will be calculated using the recommended method by Altman [73].

### Statistics for secondary outcome measures

Descriptive statistics such as mean, standard error of mean, minimum, maximum, median, and interquartile range will be calculated for all continuous variables and percentages for all categorical variables from all participants. Categorical variables will be analyzed for differences between infected and uninfected individuals utilizing Fisher’s Exact tests or the Jonckheere-Terpstra-test and confidence intervals. Continuous variables will be assessed for normality; if found to be normally distributed a t-test will be used to assess differences between infected and uninfected individuals. Multiple comparisons will be performed after data is tested for normality, normal distributed data will be analyzed using ANOVA followed by Tukey’s post hoc test, not normally distributed data will be analyzed by Kruskal-Wallis followed by Dunn´s posthoc test. If continuous variables are not normally distributed, they will be transformed to achieve normality (if a suitable transformation is available) or non-parametric statistics (Mann-Whitney-U test) will be used. For assessments of differences over time in one group (i.e. comparison to baseline values) either a paired t-test (normally distributed variables) or a Wilcoxon-signed-rank test will be used for continuous variables and the McNemar-test for related variables. 95% confidence intervals for mean, median or proportions will be calculated where appropriate.

### Model to analyze pre-post change

To evaluate if treatment of filarial infections reduces a potential effect on HOMA-IR, the pre-post change of the respective groups will be analyzed. Therefore, the individual change will be assessed for each treated participant at 12 and 18 months post treatment and will be aggregated for each group as reliable individual change. The reliable individual change will be reported together with standard effect size calculations [74]. The sample size calculation will be evaluated using a pre-post-control design [75].

### Baseline characteristics

Descriptive statistics such as numbers of participants, mean, standard error of mean, minimum, maximum, median and interquartile range will be calculated for all continuous baseline variables and percentages for all categorical baseline variables. Analyses will be conducted to determine if infected and non-infected individuals have similar characteristics at baseline. In case of a significant difference between infected individuals and non-infected individuals, Tukey’s post hoc test will be chosen for the ANOVA or Dunn´s post hoc test for the Kruskal-Wallis-test to assess differences among groups. 95% confidence intervals for mean, median or proportions will be calculated where appropriate. Further, correlations and multi-regression analysis will be used to further investigate the potential impact of the three different filarial species as well as co-infection on obesity, immune status, circulating lipid and liver enzymes. In addition, impact of the socio-economic status and life-style will be analyzed. Data will be tested for normality, if found normal, data will be analyzed using ANOVA followed by Tukey’s post hoc test and Kruskal-Wallis followed by Dunn´s post hoc test if the data was found to be not normally distributed.

### Inclusivity in global research

Additional information regarding the ethical, cultural, and scientific considerations specific to inclusivity in global research is included in the Supporting Information (S2 File).

### Potential issues

Potential issues performing this study could be the recruitment of infected patients with the indicated body mass index. Due to sustained MDA in the surrounding areas, it might prove difficult to reach the planned participant numbers for groups that include filarial infections. In addition, the global SARS-CoV2-pandemic proves to be a major obstacle in international travel and shipment of supplies. Moreover, the study sites are part of the Anglophone region which is exposed to the Anglophone crisis and social unrest, which may delay patient recruitment.

### Summary

In light of the enormous burden of novel life-style diseases including metabolic diseases and a potential beneficial effect of helminth-induced immunomodulation on those, this survey will investigate the effect of filarial immunomodulation and, importantly, also their intervention on the metabolic and inflammatory mechanisms that drive diet- and life-style-derived metabolic diseases. In case of a beneficial effect of filarial infections on the severity of metabolic diseases, this study could support the investigations that employ helminths as novel therapeutic approaches to treat autoimmune diseases and metabolic disorders driven by inflammation. Further, this survey could demonstrate the relevance of many immune modulating effects of helminths for humans consistently observed in experimental animal studies. In case of negative results regarding the worsening of metabolic symptoms or no detectable effects, this study could provide evidence against the correlation of decreased helminth infections and an emerging number of metabolic and autoimmune diseases. In both cases, crucial information about a complex microcosm of filarial-host-interactions in a human context will be obtained.

### Report of adverse events and harms

Adverse events during the trial will be listed in the CRF. Adverse events caused by the treatment will result in an interruption of the treatment and participants will drop out of the study. An experienced study doctor (TDK) will accompany the study. An interim analysis will be performed by the data management team after the 12-month follow-up.

### Dissemination policy

#### Trial results

Trial results will be communicated to the participants after the end of the study by public meetings at the site. Results of the study will undergo peer review before publication in free access journals. Raw data will be published in the aforementioned peer review journals. Diagnostic results of the infection status and potential metabolic diseases will be provided to the study participants with the next visit of the study site.

## Supporting information

S1 File (PDF)

S2 File Inclusivity in global research. (DOCX)

S3 File (PDF)

S1 Checklist SPIRIT checklist. (DOCX)
