# Supplementary material for: The design and development of a study protocol to investigate Onchocerca volvulus, Loa loa and Mansonella perstans-mediated modulation of the metabolic and immunological profile in lean and obese individuals in Cameroon
Source: PLoS One. 2023 Jun 2;18(6):e0285689. doi: 10.1371/journal.pone.0285689 (PMC10237473; doi:10.1371/journal.pone.0285689)
Supplement: S1 File — (PDF) [file pone.0285689.s001.pdf]

FIMMIP

Impact of human filarial infections on the metabolic and immunological profile

(a partially randomized, controlled, open label pilot trial)

1.1. Patient Information Sheet (English version)

This document is intended for men and women between 18 and 60 years age who live in this community and whom we are inviting to participate in a research study on influence of filarial infections (*Onchocerca volvulus, Mansonella perstans* or *Loa loa* infection) on immune and metabolic profiles.

Title of Study: Impact of human filarial infections on the metabolic and immunological profile

Principal Investigator 1 (Cameroon): Prof. Samuel Wanji

Head of Department of Microbiology and Parasitology, Faculty of Science, University of Buea, P.O. Box 63, Buea, Cameroon and Research Foundation in Tropical Diseases and the Environment (REFOTDE), P.O. Box 474, Buea, Cameroon

Phone: (237) 694727715, **E-mail:** samwandji@gmail.com

Principal Investigator 2 (Germany): Prof. Dr. rer. nat. Marc Peter Hübner

Institute for Medical Microbiology, Immunology and Parasitology (IMMIP), University Hospital Bonn, Venusberg-Campus 1, 53127, Bonn, Germany. **Phone: 0049 228 287 19177**

E-mail: huebner@uni-bonn.de

Sponsor: University of Buea, Faculty of Science, Department of Microbiology and Parasitology

We are members of a research team based in the Department of Microbiology and Parasitology at the University of Buea, Cameroon, and the Institute for Medical Microbiology, Immunology and Parasitology (IMMIP), University Hospital Bonn, Germany.

We are doing research on filarial worm infections namely *Onchocerca volvulus, Loa loa* and *Mansonella perstans* which are common in your community.

I am going to give you information and invite you to take part in this research. You do not have to decide today whether or not you will participate in the research.

There may be some words or information that you do not understand or that you have questions about. Please, feel free to interrupt me any time if you have questions and I will gladly answer them. If you have questions later, you can ask a member of our team or call the phone numbers or write e-mails to the mail-ids provided at the end of this document. Before you decide, you can talk to anyone you feel comfortable with about the research.

Background information

Type 2 diabetes is a growing challenge for health care systems in Africa. In Cameroon, more than half a million patients suffer from diabetes. Diabetes is one of the major causes for global mortality, morbidity and health care expenditures, which will aggravate especially in Africa, where around 67% of diabetic subjects are undiagnosed and it is predicted that until 2035, the number of diabetes patients will more than double.

A lot of research done by our group and other collaborators support the protective role of filarial infections and helminth-derived products on type 1 diabetes. Studies done on humans and obese animal models in the last few years supported the beneficial role of filarial infections on type 2 diabetes.

In light of the enormous global diabetes burden and the beneficial effect of helminth-induced immunomodulation on diabetes, it is important to elucidate the effect of helminth infections and their impact on diabetes.

Therefore, we aim to decipher the influence of immunomodulation during infections with the filarial nematodes *Mansonella perstans* and *Onchocerca volvulus* on glycaemic and metabolic parameters in Littoral Region, an endemic area for filarial infections.

This is the reason why we are visiting your community today. We are going to investiage the impact of filarial infections on immune and metabolic profiles to predict the risk to develop diabetes. We are approaching you because you are between 18 and 60 years of age and you live in this community where filariasis is prevalent. If you are infected by the worm or fulfil our critria to serve as an endemic control we will invite you to participate in this study.

Study Objectives

The objectives of the study are

1. To investigate the association between human filarial infections and other diseases such as diabetes, high blood pressure etc.

2. To elucidate whether treatment against filariae affect other diseases such as diabetes, high blood pressure etc.

Who can participate in this research and what examinations will we do to find out whether you can participate?

To participate in this research, you must be between 18 and 60 years of age and your body mass index (BMI) should be 25 or greater or alternatively below 25. You should not have clinical signs of tuberculosis, HIV, any known chronic disease.

To find out whether you can participate in this research, we will ask you questions about where you live, your occupation, your current medication details and about your health. We will also gather information such as your name, sex, age and diabetes history.

If you are not in good health and you need to see a doctor, we will tell you and refer you to your local health clinic.

If you are in good health we will screen you for *O. volvulus*, *M. perstans*, *Loa loa* as well as intestinal helminths. If you are positve or negative for any of the above diseases you can participate in the study if you still want to do so. After your approval, we will collect 18 ml of blood with empty stomach (last meal the evening before) in the morning and investigate your immune profile using it.

How many patients will participate in the study?

400 *O. volvulus* infected, 400 *M. perstans,* 400 *Loa loa* infected and 400 endemic controls will be involved in the study. Each group will consist of 200 obese and 200 lean individuals, as obesity is one risk factor to develop metabolic diseases including type 2 diabetes.

What will the study require?

You will participate in a study which evaluates an impact of filarial infections on immune responses and other diseases like diabetes and high blood pressure. It will take about 3 hours of your time once in 12 months. If you are infected with *M. perstans* and/or *O. volvulus* and/or co-infected with *Loa loa* you will receive treatment with the antibiotic doxycycline, you will be asked to take the drug daily for 6 weeks under supervision.

We will screen for *O. volvulus*, *M. perstans*, *Loa loa,* intestinal helminths and diabetes. If you fit into our inclusion criteria, we will collect 18ml of blood and a urine sample from you before having your breakfast to evaluate glucose level and organ function in your blood/urine. This will tell you whether you have diabetes and need treatment. This blood will be also used to determine *M. perstans* and *Loa loa* infection and microfilarial density. Blood sample collection will occur at baseline, 12 month and 18 months. For the determination of intestinal helminths we will additionally require 3 stool samples at baseline, 12 month and 18 months. To determine *Onchocerca volvulus* infection and microfilarial density, we will perform two skin snips (a superficial cut of your skin) the size of your finger tip at baseline, 12 month and 18 months. Some of your samples will be transported to the German partner institute at Bonn, Germany for further analysis. The samples will not be used for any commercial purposes but only to ensure and promote technology transfer and long-term sharing of the research results derived from those samples. In case you decide to withdraw from the study at any point during the study period, we will still keep the data collected so far and include it in the analysis.

On the day of your participation, you will be given an introduction and explanation of the study and its deliverables.

All *M. perstans, O. volvulus* and co-infected *L. loa* patients will be treated with 200mg of doxycycline daily for 6 weeks, which will eliminate the filarial infection. As the pathology in onchocerciasis is driven by the filarial offspring, doxycycline treatment will reduce pathology.

Onchocerciasis patients, *M. perstans* and *L. loa* infected patients and endemic controls will receive a single dose of 400 mg albendazole every three months with a total of 4 treatments for the elimination of intestinal helminths.

All subjects will be followed up 12 and 18 months post treatment and the immunological and disease-related parameters will be analyzed.

Am I eligible to participate in the study?

Yes, if you are infected with or without *O. volvulus, Loa loa* or *M. perstans* and if you had taken last anti-filarial therapy more than 4 months ago. Based on the information collected, the study doctor will decide if you can participate or not. If you cannot, the doctor will explain to you why.

Duration of your participation in the study

Your participation in the study will include a minimum of 3 visits in 12 month intervals for a total of three years. The visits will involve about 3 hours both for nodule palpation, skin snipping, blood glucose measurement and blood draw to determine *Loa loa* and *Mansonella perstans* microfilariae. If you are chosen for doxycycline therapy you will have a total of 46 visits, as you will have 6 weeks of daily supervised doxycycline therapy (the visits for the doxycycline therapy will be around 10 minutes each).

If you are selected for the study, you will be asked to come back in 12 and 18 months after the first sample collection which will take about 1 hour.

What are the constraints of the study?

There are no particular constraints to participate in this study.

Are there any risks in participating in the study?

We do not expect any major risks, but you could feel following discomforts:

- Needle prick for **blood drawing** and **skin snipping** to determine microfilariae include slight pain and bruising, or a small swelling on the arm at the site of needle puncture / skin sampling. The bruising may last up to 72 hours. Rarely, a swelling on the arm at

the site of needle puncture may appear which is easily treated with local pressure. Infections from the needle puncture / skin snip are rare, but if this does occur, appropriate treatment will be given. But all measures are taken to prevent these infections, by disinfecting the skin, using sterile, individual needles and covering the puncture site with a plaster. The risk of infection after a skin snip will be overcome by applying antibiotic powder on the skin.

- **Doxycycline** (the study drug) is an already marketed product for the treatment of several infectious diseases. It is an antibiotic, which has been used for decades and most side effects of this drug are well known. Reported uncommon side effects include diarrhea, nausea and discomfort on swallowing, but occur most often in people that did not eat before. Therefore, we will provide food before you receive the treatment. If, unexpectedly, you experience any side effects, report it to the research team and you will be attended by our medical team.
- It is not allowed to use the **doxycycline** during **pregnancy**, when **breastfeeding** or in children less than 8 years because it may damage developing bones. Women of child-bearing age, will therefore be tested for pregnancy and only non-pregnant women and non-breast feeding women will be enrolled for the study. Follow-up pregnancy test will be performed 2 and 4 weeks after treatment start. As doxycycline may make oral **contraceptives** (drugs taken to prevent pregnancy) less effective, you are required to use other effective methods of contraception (methods used to prevent pregnancy, including abstinence) before, during and for at least 2 weeks after the completion of the treatment. If you are a woman and found to be pregnant during the treatment phase of the study you will be counselled about the potential risks and excluded from any further participation in the treatment. However, you will continue to benefit from medical care offered by the team to the study participants. Although many commonly used drugs (including iron for anaemia, some drugs for controlling seizures and antacids for heartburn) may make the study drug less effective, the study drug has little effect on other drugs with the exception of iron. Therefore, you will be asked about any medication you are taking and advised if necessary.
- In the event that you experience any **side-effect** of the study drug during the course of this study, you should immediately contact the doctor-in-charge of the study, who will determine what action to take. You will be informed of any new important knowledge

about the drugs used in the study that may lead to you changing your mind about continuing. It is well possible that there will not be any side-effects but you need to know that they might occur.

- **For women of child-bearing age.** All drugs are potentially dangerous to the unborn child. This is why you can only be included in the study if you are not pregnant and are using effective pregnancy-preventing methods. To avoid unnecessary risks, and with your consent, you will undergo a pregnancy test before you can be included in the study. You have to be aware that you should not get pregnant before, during and at least 2 weeks after doxycycline treatment because the treatment could harm your unborn child. Therefore you should use an effective barrier method of pregnancy prevention such as condoms, or other preventive methods including abstinence. If you are already taking birth control pills by mouth, you should use a barrier method of birth control in addition for the duration of the study treatment period since the efficiency of birth control pills may be reduced. Appropriate advice will be given during the consent and the process prior to your addition and supplies may be provided should you request them. The pregnancy test will be repeated every 2 weeks until end of treatment. If you think you have become pregnant during the study and especially during the treatment period, you should inform the study doctor immediately. If you do become pregnant during the treatment period, you will be withdrawn immediately from treatment and followed up as appropriate. The study doctor (on behalf of the study sponsors) will follow up the progress of your pregnancy until after the birth of the baby.
- **Albendazole** is a well-known and safe drug used for the treatment filarial infections. Reported uncommon side effects include headaches, minor gastrointestinal problems skin rashes and itchiness.

Am I insured in the event of any unforeseeable damages or accidents?

The Sponsor has taken out an insurance policy that will cover you if you are injured as a result of taking part in the study. The patients who participate in the assessments will be covered by insurance against the risk of accidents involving injury or damage that occurs during the study. If you are in doubt, just like in case of discomfort or undesirable effects

related to your participation in the study, please, contact the principal investigator or the other investigators of this study directly.

Will my participation in the study benefit other patients with filariasis?

Your participation in this study will help us to understand the association between helminth infections and diseases such as diabetes. If the study is a success, this will lead us to identify protective mechanisms leading to potential new therapeutic measurements to control diabetes.

Will I be paid and/or pay for the diagnosis?

- You do not have to pay anything to participate in the study.
- Your diagnosis of *O. volvulus*, *Loa loa, Mansonella perstans*, diabetes and dyslipidemia will be done free of charge
- You will know your glycemic status, lipid profiles
- Depending on the site, your travel expenses will be paid directly by the centre or a car will collect you and drive you back home.
- In case that you will be diagnosed with diabetes, we will refer you to a specialist, who will advise you on the diet and help you.

Am I free to decide whether I will participate or not?

Participation in this study is completely voluntary. You can refuse to take part in the study at any time and your decision will have no effect on your relations with the study investigators.

Can my participation be stopped?

Your participation in this study may be stopped at any time by:

- The Principal Investigators, the Sponsor or the Health Authorities for breaching any of the study inclusion criteria
- The study doctor, for medical reasons
- Yourself, without having to justify your decision

However, for your participation to be useful to the study, it is important that you remain in the study and that you attend all of the scheduled meetings.

How will my personal and medical information be used?

All data will be collected and stored safely and reported in a pseudonymous form. Only the Principal Investigators, the team involved in the study, the Sponsor of the study and/or members of the *Comité National d'Ethique de la Recherche pour la Santé Humaine** (CNERSH) will have access to the original data under strict confidentiality.

Will I know about the results of the study?

We will inform you of the results of the examination for palpable nodules, the skin snip, diabetes status, glycemic load and lipid profiles.

After the end of the research, meetings will be held in the community and the study results will be presented.

Does this study comply with current laws and regulations?

This study will be conducted in accordance with the laws and regulations of your country.

Who should I contact if I have further questions on the study?

- You can telephone any time personally meet and speak to Professor Samuel Wanji if you have any questions about the research study. His telephone number is (237) 694 727 715.

- You can also contact the *Comité National d’Ethique de la Recherche pour la Santé Humaine* (CNERSH), who approved the plan of our research study. If you want additional information about the Committee or if you want to ask questions about the study and your rights as a participant, you can contact the Committee by calling (237) 227 621 14.

* National Ethics Committee for Research on Human Health

1.2. Consent Form for Study (English version)

Impact of human filarial infections on the metabolic and immunological profile

I, the undersigned Mr/Ms ____, residing in ______________________________________ community, certify that I have read the information above concerning the objectives and conduct of the study by the team of Professor Samuel Wanji from University of Buea, or that the information was read to me. I have had the opportunity to ask questions and all of the questions I asked were answered. I agree to participate in the study and I know that I can withdraw from the study any time I want. I have received a copy of the information document.

Signature of participant: ________________________________

Date: ___________________ Place: _____________________

If the participant is potentially illiterate

I, the undersigned ______________________________ hereby testify to have followed the accurate reading of the information on the study to the potential participant and that he/she has had the opportunity to ask questions and has received a copy of the information document.

I confirm that ____ consented freely.

Signature of witness: ____________________________________

Fingerprint participant: ___________________________________

Date: _____________________ Place: _________________________

Name of member of the study team**:** ____

Signature of team member: _______________________ Date: _______________

Date of Interview: ☐☐ / ☐☐ / ☐☐☐☐

Day Month Year

Name:

Father's Name:

**Address (Home)**	**Address (Work Place)**
______________________	______________________
______________________	______________________
____________	____________
Postal code: ________	Postal code: ________
Land Line: ________________	Land Line: ________________
Mobile: ________________	Mobile: ________________
Email: ________________	Email: ________________

For follow-up, please note down the details of one close relative or friend, who can be contacted, if the subject moves from the area

Name: ____

Relationship: ____

Address/Details:

____

____

____

Postal code: ______________

Landline number with Area code: ____________________

Mobile number: ____________________

Email ____________________

Contact details of Village authority / Village headman:

If the participant is below 21 years of age

Do you understand the Patient Information Sheet and the consent form? ☐ yes ☐ no

Did you have enough time for your personal decision? ☐ yes ☐ no

Do you understand that blood will be drawn during this study? ☐ yes ☐ no

Can you refuse to participate in the study at any time? ☐ yes ☐ no

Are you forced to participate in this observational study? ☐ yes ☐ no

Do you have any questions? ☐ yes ☐ no

If yes, documentation of questions:

____

____

____

____

Do you know who to call if you have questions? ☐ yes ☐ no

Name of the participant (in block letters): ___________________________________

Date of birth (year of birth, if exact date is unknown): |__|__| / |__|__| / |__|__|__|__|
(dd / mm / yyyy)

Indiv. No.: CM – |__|__|__| - |__|__|__|
(village code) - (consecutive patient no)

I hereby certify that the contents of the Patient Information Sheet and the Informed Consent Form have been read by me/ interpreted and explained to me and my parents in detail.
By:____
(Name of investigator in block letters)

I fully understand the content of the Patient Information Sheet and the Informed Consent Form. The information given to me has permitted me to make a fully informed and free decision about my participation in the observational study. I am aware that my participation is voluntary and that I can withdraw at any time and for any reason without penalty or loss of benefits. By signing this consent form, I do not waive any legal rights, and the investigator(s) or sponsor are not relieved of any liability they may have.

I thereby append my signature/mark (right thumbprint) to this Informed Consent Form, as evidence of my agreement to participate in all procedures required for the clinical trial.

Date: |__|__| / |__|__| / |__|__|__|__|
(dd / mm / yyyy)

If needed, thumbprint of participant:

____
(Participant's signature)

____
(Participant's name in block letters)

Parent's signature

I hereby certify that I was present when the contents of the Patient Information Sheet and the Informed Consent Form were read/interpreted and explained to my son/daughter

____

(Name of participant in block letters)

He/she seems to have fully understood the contents of the Patient Information Sheet and the Informed Consent Form before appending his/her signature or making his/her mark (right thumb print) to this Informed Consent Form. As the legal representative I hereby agree with the participation.

Date: |__|__| / |__|__| / |__|__|__|__|
(dd / mm / yyyy)

If needed, thumbprint of parent:

____

(Signature of the parent)

____

(Name of the parent in block letters)

WITNESS (independent from research team)

I hereby certify that I was present when the contents of the Patient Information Sheet and the Informed Consent Form were read/ interpreted and to

____

(Name of participant in block letters)

and his/her legal representative.

He/she and his/her legal representative seem to have fully understood the contents of the Patient Information Sheet and the Informed Consent Form before appending their signatures or making their marks (right thumb print) to this Informed Consent Form in my presence as evidence of their agreement to participate in the above mentioned clinical trial.

Date: |__|__| / |__|__| / |__|__|__|__|
(dd / mm / yyyy)

____

(Signature of the witness)

____

(Name of the witness in block letters)

INFORMANT (Investigator)

I hereby certify that the contents of the Patient Information Sheet and the Informed Consent Form were interpreted and explained by me to

____

(Name of participant in block letters)

and his/her legal representative.

He/she and his/her legal representative seem to have fully understood the contents of the Patient Information Sheet and the Informed Consent Form before appending their signatures or making their marks (right thumb print) to this Informed Consent Form in my presence as evidence of their agreement to participate in the above mentioned clinical trial.

Date: |__|__| / |__|__| / |__|__|__|__|

(dd / mm / yyyy)

____

(Signature of the investigator)

____

(Name of the investigator in block letters)
