# Supplementary material for: The design and development of a study protocol to investigate Onchocerca volvulus, Loa loa and Mansonella perstans-mediated modulation of the metabolic and immunological profile in lean and obese individuals in Cameroon
Source: PLoS One. 2023 Jun 2;18(6):e0285689. doi: 10.1371/journal.pone.0285689 (PMC10237473; doi:10.1371/journal.pone.0285689)
Supplement: S3 File — (PDF) [file pone.0285689.s003.pdf]

Study Protocol for Ethical Approval

Impact of human filarial infections on the metabolic and immunological profile

**Version:**	**4.3; March 2023**
**Acronym:**	**FIMMIP**
**Study centre:**	Department of Microbiology and Parasitology (DMP), University of Buea, Buea, Cameroon
**Study design:**	Cross-sectional study
**Study area:**	Littoral region in Cameroon
**Funding:**	DFG German-African Cooperation Projects in Infectology
**Grant Number:**	
**Proposed start date:**	December 2019
**Proposed end date:**	December 2026

Table of Contents

1 PRINCIPAL INVESTIGATORS' STATEMENT 4

2 TRIAL ADMINISTRATION STRUCTURE 5

2.1 Principal investigators / contact details 5

2.2 Investigators 5

2.3 Sponsor Details 6

3 PROTOCOL SYNOPSIS 7

4 INTRODUCTION 9

4.1 Summary 9

4.2 Background 9

4.3 Rationale 10

5 OBJECTIVES 11

5.1 Primary objective 11

5.2 Secondary objective 11

6 STUDY DESIGN 11

6.1 Study site 11

6.2 Estimated duration of the study 11

6.3 Group allocation 12

6.4 Outcomes and variables 12

6.5 Analysis whether termination of *Mansonella* and Onchocerciasis infection via 6-week Doxycycline (200mg per day) treatment will change the metabolic and immunological profiles (analysis according to the descriptions above) 12 and 18 months post treatment. Overall timeline of the study 13

7 SELECTION AND INCLUSION OF VOLUNTEERS 13

7.1 Inclusion criteria 13

7.2 Exclusion criteria 14

8 STUDY PROCEDURES 14

8.1 Informed Consent / Patient enrolment 15

8.2 Clinical examination and laboratory analyses 15

9 SAMPLE COLLECTION AND DATA MONITORING 16

9.1 Study monitoring 16

9.2 Sample collection: skin biopsies and blood taking 16

9.3 Intervention pilot study 17

9.3.1 Side effects of doxycycline: 18

9.3.2 Precautions to mitigate drug specific risks (Doxycycline) ... 19
9.3.5 Adverse events ... 21
9.4 Assessment of immunological parameters ... 21
9.5 Source documentation ... 21
9.6 Study documentation, data safety and confidentiality ... 22
9.7 Data Collection & Management ... 22
9.8 Retention of records ... 23
9.9 Protocol revisions ... 23
9.10 Sample transport from Cameroon to Bonn ... 23
9.11 Termination of the trial for individual subjects ... 24
9.11.1 Termination by the participant ... 24
9.11.2 Termination by the investigator ... 24
9.12 Study Monitoring ... 24
10 STATISTICAL CONSIDERATIONS ... 25
10.1 Sample size justification and statistical analysis ... 25
11 DELIVERABLES ... 25
12 RESULTS ... 25
13 HUMAN RIGHTS PROTECTION ... 26
13.1 Research Ethics Committee ... 26
13.2 Informed Consent Forms (ICF) ... 26
13.3 Benefits ... 26
13.4 Risks ... 26
13.5 Confidentiality ... 27
13.6 Compensation & patient insurance ... 27
13.7 Study Registration ... 27
13.8 Data safety and monitoring committee (DMSC) ... 27
14 LIST OF ABBREVIATIONS ... 27
15 REFERENCES ... 28

ANNEX ICF Informed Consent Form + Patient Information Sheet
ANNEX CRF Case Report Form

1 PRINCIPAL INVESTIGATORS' STATEMENT

I, the undersigned, have reviewed this protocol and relevant appendices and agree to conduct the designed study as described herein. In addition, I agree to adhere to the principles of the current International Conference on Harmonisation - Good Clinical Practices (ICH-GCP).

Prof. Dr. rer. nat. Marc P. Hübner, Institute for Medical Microbiology, Immunology and Parasitology (IMMIP), University Hospital Bonn, Germany

Signature: Date: March 28th, 2023

I, the undersigned, have reviewed this protocol and relevant appendices and agree to conduct the designed study as described herein. In addition, I agree to adhere to the principles of the current International Conference on Harmonisation - Good Clinical Practices (ICH-GCP).

Prof. Dr. Samuel Wanji, Department of Microbiology and Parasitology (DMP), University of Buea, Buea, Cameroon

Signature: Date: March 28th, 2023

2 TRIAL ADMINISTRATION STRUCTURE

2.1 Principal investigators / contact details

Cameroon

Prof. Dr. Samuel Wanji, PhD
Department of Microbiology and Parasitology (DMP),
University of Buea,
Buea, Cameroon
Tel. 00(237)694727715
E-mail: samwandji@gmail.com

Germany

Prof. Dr. rer. nat. Marc Peter Hübner
Institute for Medical Microbiology,
Immunology and Parasitology (IMMIP),
University Hospital of Bonn,
Venusberg-Campus 1, 53127, Bonn
Tel: 0049 228 287 19177
E-mail: huebner@uni-bonn.de

Prof. Dr. med. Achim Hoerauf
Institute for Medical Microbiology,
Immunology and Parasitology (IMMIP),
University Hospital of Bonn,
Venusberg-Campus 1, 53127, Bonn
Tel: 0049 228 287 15675
E-mail: Hoerauf@uni-bonn.de

2.2 Investigators

Cameroon

Dr. Jerome Fru, PhD

Department of Microbiology and Parasitology, University of Buea and Research Foundation for Tropical Diseases and the Environment Buea (REFOTDE)
Tel: (00237) 678072044

E-mail: jerome.frucho.1@gmail.com

Ms Ayuk Elizabeth, PhD student

Department of Microbiology and Parasitology, University of Buea and Research Foundation for Tropical Diseases and the Environment Buea (REFOTDE)

Tel: +237 675138057

E mail: manyiwhales@yahoo.com

Ngong Innocentia Nji, PhD student

Department of Microbiology and Parasitology, University of Buea and Research Foundation for Tropical Diseases and the Environment Buea (REFOTDE)

Tel : +237 679459667

E mail: innocentiafrucho@gmail.com

Germany

Benjamin Lenz, PhD student
Dr. Manuel Ritter
Dr. Indulekha Karunakaran, PhD
Dr. Alexandra Ehrens, PhD student
Institute of Medical Microbiology, Immunology and Parasitology (IMMIP),
University Hospital Bonn
Venusberg-Campus 1
53127 Bonn, Germany
Tel: +49-228-287-19169
Fax: +49-228-287-19573

2.3 Sponsor Details

Cameroon **University of Buea, Faculty of Science, Department of Microbiology and Parasitology**

3 PROTOCOL SYNOPSIS

**Protocol Title**	Impact of human filarial infections on the metabolic and immunological profile
**Participants**	Healthy male and female volunteers:<br>• aged 18-60 years<br>• BMI ≥25, <25<br>• last intake of ivermectin (IVM) more than 4 months ago<br>• last intake of anti-filarial antibiotic treatments more than 12 months ago<br>• Residents of the endemic area for at least 5 years
**Number of participants**	1200 included in study (approx. 5000 will be screened)
**Study design**	Interventional ~~randomized controlled~~ open label pilot trial
**Study groups**	Group 1: *Mansonella* patients<br>• Positive for *Mansonella perstans*<br>• Microfilariae (Mf) positive and/or *M. perstans* PCR positive<br>• BMI ≥25 (n =200)<br>• BMI <25 (n =200)<br>Group 2: Onchocerciasis patients<br>• Positive for *Onchocerca volvulus*<br>• Microfilariae (Mf) positive and/or *O. volvulus* PCR positive<br>• BMI ≥25(n =200)<br>• BMI <25 (n =200)<br>Group 3: Endemic normal<br>• Mf negative in skin and blood<br>• *O. volvulus* and *M. perstans* PCR negative<br>• Negative for other filarial and helminth infections<br>• Normal range of eosinophils and IgE levels<br>• BMI ≥25 (n =200)<br>• BMI <25 (n =200)<br><br>Group 4: Loiasis patients<br>• Positive for *Loa loa*<br>• Microfilariae (Mf) positive and/or *Loa loa* PCR positive<br>• BMI ≥25(n =200)<br>• BMI <25 (n =200)
**Treatment**	<u>Doxycycline:</u><br>• All individuals of group 1 and 2 as well as double infected individuals (*Loa loa* in addition to either *O. volvulus* or *M. perstans*) will be selected for doxycycline therapy<br>• Daily doxycycline therapy for 6 weeks with 200mg<br>• Individuals with <50kg will receive 100mg doxycycline daily for 6 weeks<br>• Individuals with <40kg are excluded

	Albendazole:<br>• Community based mass drug administration for group 1, 2, 3 and 4<br>• Individuals will receive one dose of 400mg Albendazole every three months with a total of 4 treatments
**Time period**	2019-2026

4 INTRODUCTION

4.1 Summary

Parasitic filarial nematodes modulate the host's immune system to enable their long-term survival within the host. This immunomodulation by helminths impacts the immune response of the host to bystander antigens and mitigates allergies and autoimmune diseases by suppressing associated pro-inflammatory immune responses. Such chronic, low grade pro-inflammatory immune responses also occur during obesity and are an important etiological factor for the pathogenesis of type 2 diabetes (T2D). Recently, experimental animal studies from our lab demonstrated that infections with the filarial nematode *Litomosoides sigmodontis* have a beneficial impact on diet-induced glucose intolerance and a similar protective effect was observed for other helminth infections in experimental animal studies and cross-sectional human studies. In this proposal, we aim to analyze the impact of human filarial infections *Onchocerca volvulus*, *Loa loa* and *Mansonella perstans* on the metabolic profile in association with changes in the systemic soluble and cellular immune response. In this regard, we will analyze glycaemia, lipid profile, liver function enzymes, adipocytokines, pancreatic and gut hormones, and the level of insulin resistance in obese individuals and compare it to lean individuals. A detailed analysis of the peripheral blood cell composition, intracellular cytokine-producing cells, cytokines/chemokines in plasma and released after *in vitro* whole blood stimulation, as well as total and filariae-specific humoral responses will be performed. Those responses will be compared to obese and lean endemic controls and followed-up after anti-filarial treatment with doxycycline for the *Mansonella* patients and onchocerciasis patients. All participants including endemic controls and *Loa loa* infected individuals will receive 4 rounds of albendazole.

4.2 Background

Parasitic helminth infections remain a public health problem in developing countries of the tropics and subtropics. Infections with *Onchocerca volvulus* can cause severe dermatitis, visual impairment and vision loss and it is estimated that approx. 17 million people are currently infected with *O. volvulus*, with 99% of all cases occurring in sub-Saharan Africa. *Mansonella perstans* on the other hand leads to infections that are often clinically silent and it is estimated that 120 million people are infected with *M. perstans*. Previous studies in Cameroon, which were in part done in collaboration between the two partner institutes (IMMIP, University Hospital of Bonn and the DMP, University of Buea) as part of a different DFG-Africa infectology project (Map2Co), revealed that infections with those helminths are endemic in the study area used in Cameroon.

Helminths generally induce in their hosts type 2 immune responses that are characterized by elevated type 2 cytokines, increased levels of IgE and IgG4 as well as an eosinophilia. Over time, helminths establish regulatory responses via the expansion of alternatively activated macrophages, regulatory T cells and the release of anti-inflammatory IL-10 & TGFβ in their hosts that enable their long-term survival in the human host, but also impact bystander immune responses. Previously, several human and experimental animal studies demonstrated that helminth infections prevent or ameliorate dysregulated immune diseases like autoimmune diseases, sepsis and allergies. A similar protective effect was recently suggested for metabolic diseases like T2D (1-11).

T2D is a growing challenge for health care systems in Africa. 80% of diabetes cases occur in low and middle income countries (12) and the largest increase of diabetes cases is expected to take place in regions with developing economies due to associated changes in life style and environment (12). Lack of awareness, health

care facilities and expenditure in underdeveloped countries has led to diabetes becoming a major problem. In Cameroon more than half a million patients suffer from diabetes. However, their diabetes related expenditure amounts to only 123 US$/person in comparison to >5000 US$/person in Germany (12). These figures strongly claim that diabetes is one of the major causes for global mortality, morbidity and health care expenditures, which will aggravate especially in Africa, where around 67% of diabetic subjects are undiagnosed and it is predicted that until 2035, the number of diabetes patients will more than double (12).

Several experimental studies by our group and colleagues support the protective role of helminth infections and helminth-derived products on type 1 diabetes (13-17). Obesity-induced inflammation is a primary etiological factor for the pathogenesis of T2D (18-20) and cell types associated with type 1 inflammation like classically activated macrophages, Th1 cells, neutrophils, NK cells and CD8 T cells are implemented in the pathogenesis of insulin resistance (21-26). Thus, helminth-induced type 2 and regulatory immune responses may counteract type 1 driven inflammation and it can be speculated that helminth infections have a beneficial impact on metabolic diseases such as T2D. Cross-sectional studies on humans and mechanistic studies on obese animal models published in the last few years suggested such a beneficial role of helminth infections on T2D. For instance, an epidemiological study in south India showed that prevalence of lymphatic filariasis decreased with the progression of glucose intolerance (1). Similarly, a history of *Schistosoma* infection was negatively associated with several glycemic parameters in a cross-sectional study in a rural community in China (2). A negative association between diabetes prevalence and *Strongyloides stercoralis* infection was observed in Australian Aboriginals (3). In Flores Island, Indonesia, individuals with soil transmitted helminth infections had a lower homeostasis model assessment of insulin resistance (HOMA-IR) and body mass index (BMI) (4).

Consistent with these human studies, a series of mechanistic studies in diet-induced obese mice revealed that helminth infections/helminth derived product administration induced protective type 2 immune responses, expanded eosinophils, ILC2s and AAM (5-7). This correlated with reduced glucose intolerance, hepatic steatosis and in some cases decrement in body weight and adipose tissue mass (8). Identified protective immune responses included the mitigation of adipose inflammation partly through IL-10 and suppressed hepatic lipogenesis (9), the triggering of the IL-33/ILC2 axis (10), induction of eosinophil and AAM expenditure in adipose tissue (6) and eventually stimulation of adipose tissue browning as type 2 immune responses are a promoter of browning, which increases energy expenditure (13, 27).

Besides the mentioned cross-sectional reports on humans and mechanistic studies on animal models, there is no longitudinal follow-up study that investigated the causal link between helminth infections, immunomodulation and metabolic responses. In light of the enormous global diabetes burden and the beneficial effect of helminth-induced immunomodulation on diabetes, it is important to elucidate the effect of helminth infections and their eradication on diabetes. Therefore, we aim to decipher the influence of immunomodulation by infections with the filarial nematodes *Mansonella perstans*, *Loa loa* and *Onchocerca volvulus* on glycemic and metabolic parameters in Southwest Cameroon, an endemic area for filarial infections.

4.3 Rationale

Clinical immunological studies in obese humans will decipher the impact of infections with the filariae *Mansonella perstans, Loa loa* and *Onchocerca volvulus* on glycaemic and metabolic parameters and associate

those with the systemic immune profile. Comparison to lean infected individuals as well as obese and lean endemic controls and follow-up analyses after anti-filarial treatment will determine the longevity of the filarial immunomodulation and indicate whether MDA programs to eliminate helminth infections may facilitate the development of T2D.

In brief, blood samples from *Mansonella* patients (Group 1), onchocerciasis patients (Group 2), *Onchocerca* and infection-free endemic individuals (Group 3) and *Loa loa* infected individuals will be assessed for glycemic and metabolic parameters and immune profiling will be performed. Each group will consist of 200 lean and 200 obese individuals. We aim to identify if there is

i) an association between filarial infection, immunological responses and the glycaemic and metabolic profile and
ii) whether anti-filarial treatment alters the immunological responses and the glycaemic and metabolic profile.

5 OBJECTIVES

5.1 Primary objective

Investigation for associations between a human filarial infection, the systemic immune response as well as the metabolic and glycemic profile

5.2 Secondary objective

The longevity of the filarial immunomodulation and its impact on the systemic immune response also on the metabolic and glycemic profile will be determined following anti-filarial treatment.

6 STUDY DESIGN

6.1 Study site

Within this proposed study a cohort will be collected, consisting of lean and obese endemic controls (group 3) and lean and obese individuals infected with either *M. perstans*, *Loa loa* or *O. volvulus* (groups 1,2 and 4). This study will be conducted in the Littoral region of Cameroon, an endemic area of *Onchocerca volvulus*, *Loa loa* and *Mansonella perstans*.

6.2 Estimated duration of the study

Recruitment of patients and sample collection will begin as soon as the ethical clearance of the revised protocol has been obtained with the aim of beginning in December 2019 as the funds have already been received from the DFG. Treatments with doxycycline and albendazole will be performed as soon as the ethical clearance of the newly revised protocol was obtained. The study comprises several stages, consisting of information of the regional health authorities, districts and communities, recruitment of volunteers, sample collection, sample processing, sample transport/export, data collection and analysis followed by preparation of publications. The patients will be followed for a period of 3 years, in 12-month intervals. It is estimated that the study will run until the end of December 2026.

6.3 Group allocation

Volunteers of both sexes (stratified according to age, gender and socio-economic status) with a BMI above ≥30 (obese) and <25 (lean), will be assigned to the following groups according to the clinical assessment (Fig. 1):

- 200 obese and 200 lean individuals with onchocerciasis (presence of microfilariae in the skin, *O. volvulus* PCR positive). All onchocerciasis patients will be treated with 200mg doxycycline daily for 6 weeks. They will also receive single dose of 400 mg albendazole every three months with a total of 4 treatments. *M. perstans*, *O. volvulus* as well as *Loa loa* co-infected individuals will be included and treated with doxycycline and albendazole, in case that not sufficient individuals with *O. volvulus* infection alone are identified.
- 200 obese and 200 lean individuals with *M. perstans* (presence of microfilariae in the blood, PCR positive). All *M. perstans* infected obese and lean patients will be randomly selected for anti-filarial therapy with 200mg of doxycycline daily for 6 weeks. They will also receive single dose of 400 mg albendazole every three months with a total of 4 treatments. *M. perstans* and *O. volvulus* as well as *Loa loa* co-infected individuals will be included and treated with doxycycline and albendazole, in case that not sufficient individuals with *M. perstans* infection alone are identified.

- 200 obese and 200 lean *Loa loa* infected and 200 obese and 200 lean healthy endemic individuals without onchocerciasis, *Mansonella perstans* infection with normal eosinophil counts and IgE levels. All endemic individuals will receive single dose of 400 mg albendazole every three months with a total of 4 treatments.

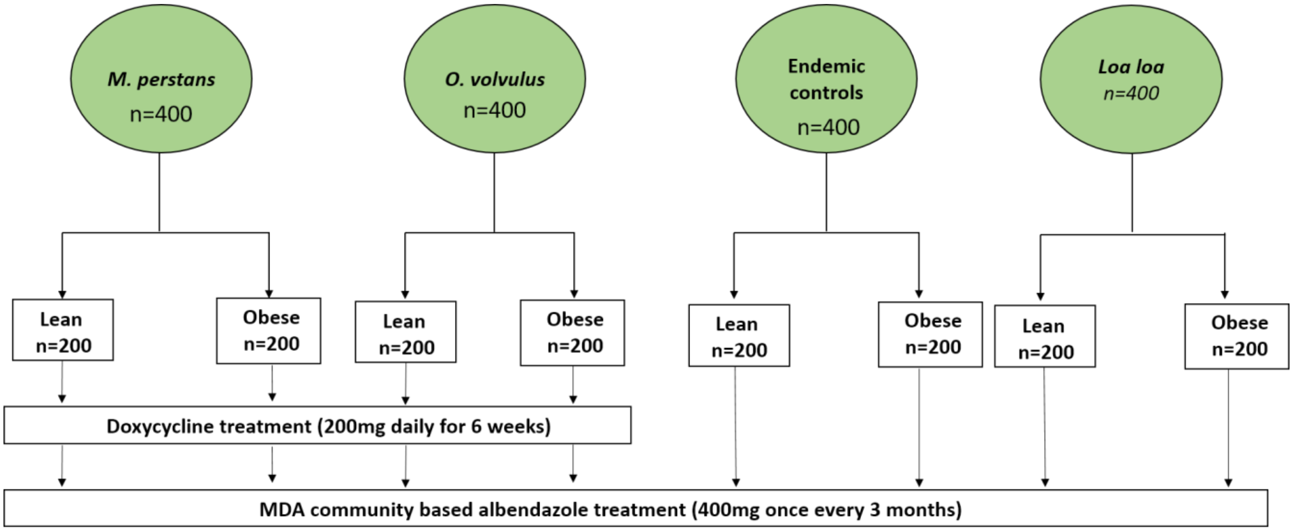

Fig. 1: Distribution of study participants

6.4 Outcomes and variables

Determination of fasting blood glucose levels, glycated hemoglobin (HbA1c), total cholesterol, triglycerides, and high density lipoprotein cholesterol (HDL-C), low-density lipoprotein cholesterol (LDL-C) and liver function enzymes-AST (aspartate aminotransferase), ALT (alanine aminotransferase), alkaline phosphatase (ALP), and γ-glutamyltranspeptidase (GGT), adipokines (adiponectin, leptin, visfatin, resistin, and

plasminogen activator inhibitor-1 (PAI-1)), pancreatic hormones (Glucagon, Insulin and C-peptide) and gut hormones (ghrelin, glucose-dependent insulinotropic polypeptide (GIP) and glucagon-like peptide 1 (GLP-1)). To assess kidney function, serum creatinine, urea and urine albumin will be measured. Albumin concentrations will be measured in fasting urine samples. Microalbuminuria and macroalbuminuria will be diagnosed if the albumin excretion is between 30 and 299 µg/mg and was ≥300 mg/g of creatinine, respectively. We will assess the impact of filarial infection on gut leakage during obesity by screening the plasma for the microbial translocation markers LPS-binding protein (LBP), endogenous endotoxin-core antibodies (EndoCAb), systemic LPS levels and intestinal fatty acid-binding protein (iFABP). Acute-phase proteins alpha-2-macroglobulin (α-2M), C-reactive protein (CRP), haptoglobin, and serum amyloid A protein (SAA).

- Insulin resistance calculated based on HOMA-IR
- Total and differential leucocyte counts as well as absolute and relative quantification of naïve, effector and central memory CD4+ and CD8+ T cells, regulatory T cells, NK cells, subset of monocytes, dendritic cells and B cells in whole blood via flow cytometry in a subset of n=30 in each group
- Frequencies of intracellular cytokine-producing cells of in vitro whole blood cell cultures in response to PMA/ionomycin, anti-CD3/anti-CD28, Pam3Cys, LPS, or palmitic acid determined by flow cytometry in a subset of n=30 in each group
- Pro- and anti-inflammatory cytokines and chemokines in plasma, whole blood culture supernatants analysed by multiplex cytokine assays
- Total humoral immune responses in serum IgG1, IgG2, IgG3, IgG4, IgA, and IgE and antibody titres against parasite antigens quantified by ELISA
- Microfilariae burden in *Mansonella*, *Loa loa* and *O. volvulus* patients.
- Height, weight, waist circumference, body fat, blood pressure and electrocardiogram using standard techniques. Calculation of BMI
- Analysis of the above mentioned parameters over a period of 3 years, in 6-month intervals

6.5 Analysis whether termination of *Mansonella* and Onchocerciasis infection via 6-week Doxycycline (200mg per day) treatment will change the metabolic and immunological profiles (analysis according to the descriptions above) 12 and 18 months post treatment. Overall timeline of the study

Patient recruitment & anti-filarial therapy

Follow-up 1 – 12 months after treatment onset

Follow-up 2 – 18 months after treatment onset

7 SELECTION AND INCLUSION OF VOLUNTEERS

7.1 Inclusion criteria

Patients eligible for the study must comply with all of the following:

- Participants will be male and female between 18-60 years old
- BMI equal to or above 25 or below 25
- Body weight > 40kg
- Last intake of ivermectin at least 4 months ago
- Last intake of anti-filarial antibiotic treatment more than 12 months ago
- Resident in the endemic study area for at least 5 years

- *O. volvulus* patients, microfilariae skin snip positive and/or PCR positive for *O. volvulus*
- *M. perstans* or *Loa loa* patients positive for microfilariae and/or PCR
- Endemic controls, judged by absence of microfilariae, palpable onchocercoma, PCR negative for *M. perstans* and *O. volvulus*. Individuals should be free of other helminth infections and possess normal eosinophil frequencies (0.5-4%).
- Good general health without any clinical condition requiring long-term medication
- Normal white blood cell counts (3,5-11,3 x 10^3/µl)
- Willingness to participate in the study as evidenced by signing the Informed Consent Form or parental signature in the ICF for subjects less than 21 years

7.2 Exclusion criteria

Patients are ineligible to participate in the study, if they have any of the following:

- Pregnancy (dipstick pregnancy test)
- Lactating mothers
- Last intake of ivermectin (IVM) less than 4 months ago
- Intake of anti-filarial antibiotic treatment (tetracycline) less than 12 months ago
- Evidence of tuberculosis (clinical aspects)
- Evidence of clinical aspects of HIV infection
- Evidence/previous diagnosis of chronic diseases (urolithiasis, liver cirrhosis, congestive heart failure, chronic lung diseases, chronic infections other than filariae, viral hepatitis)
- Evidence of autoimmune diseases and allergies
- Evidence of acute infection (haematuria, cough, fever). Evidence of clinically significant neurological, cardiac, pulmonary, metabolic, rheumatologic or renal disease as far as can be assessed by history of individuals, physical examination, and/or laboratory examinations
- Childbearing potential and not willing or able to use methods to prevent a pregnancy for the entire treatment duration in addition to hormonal contraception (e.g. condoms) unless surgically sterilized/ hysterectomized or any other criteria considered sufficiently reliable by the investigator
- Behavioural, cognitive or psychiatric disease that in the opinion of the trial clinician affects the ability of the participant to understand and cooperate with the study protocol
- Laboratory values which will lead to exclusion
    1. Haemoglobin < 8 g/dL
    2. Neutrophil count <500/µl*
    3. Platelet count <100 000/µl
    4. Creatinine > 2 times upper limit of normal
    5. AST (GOT) > 2 times upper limit of normal
    6. ALT (GPT) > 2 times upper limit of normal
    7. γ-GT > 2 times upper limit of normal
    8. HbA1c above 44mmol/mol Hb (6%)

    *The neutrophil exclusion criteria was adjusted due to the obtained data from 2000 screened participants. All participants displayed a decreased mean neutrophil blood level independent if infection status and general health indicating a regional trend towards reduced neutrophil numbers. This circumstance for the African population was described previously [34-37].

8 STUDY PROCEDURES

Ethical approval has already been obtained from the Institutional Review Board of the Faculty of Health Science of the University of Buea, the Cameroon National Ethical Committee

(No2019/03/1153CE/CNERSH/SP) and the Ethical Commission at the University of Bonn (Germany) (Lfd.Nr.046/18). An agreement with local authorities in Littoral region and the community chiefs and elders of the participating villages will also be obtained.

8.1 Informed Consent / Patient enrolment

The study will be explained in detail to the subjects in the local language (Pidgin or French). Each participant will be informed of the voluntary nature of the study and be encouraged to ask questions throughout the duration. Every volunteer will demonstrate the willingness to participate by signature, written or thumbprint on the consent form (see Annex ICF). Following recruitment, patients meeting the entry criteria will be enrolled in the study. Study subjects will be allowed to withdraw from the study at any point of the study. Recruitment will be directly carried out in the villages. Before start of recruitment the research team will visit the village elders to explain the planned study in detail and a pre-screening for age and sex, health, medication status, BMI and infection will occur. At the next visit all villagers will be invited to come to a public meeting where the study will again be explained in detail. After this meeting all interested volunteers, who are between 18 and 60 years old and signed the Informed Consent Form, will be invited for the first screening. In case of subjects below 21 years ICF will be signed by the parents. The screening will be carried out by trained members of the research team under the supervision of Investigators. A medical doctor will be present to do the physical examination and to take the medical history of the volunteers. The blood sampling and ECG will be done by trained and qualified research personnel. The screening will be carried out directly in the villages. The research team will use rooms provided by the village or will bring tents that guarantee the privacy of the volunteers during examination. All volunteers who proved to be eligible for the study during the screening visit will be invited to come to the enrolment which will also be carried out directly in the village.

All patient data will be kept confidential and it will be explained that the samples will only be used for the study defined in the consent form (see Annex ICF). Some of the samples will be transported to the German partner institute at Bonn, Germany for further analysis. The samples will not be used for any commercial purposes but only to ensure and promote technology transfer and long-term sharing of the research results derived from those samples. In case the participant decides to withdraw from the study at any point during the study period, the data collected till far from the participant will be kept and included in the analysis.

8.2 Clinical examination and laboratory analyses

Clinical examinations (Annex CRF) include:

- Medical history including a questionnaire to assess demographic data, socioeconomic factors, educational factors, and behaviour during enrolment
- Skin biopsies (2 skin biopsies of 2 mm diameter at the posterior iliac crest) to assess microfilaridermia during screening, follow-up 1 and follow-up 2
- Urine for dipstick tests (pregnancy test and urine dipstick for haematuria, proteinuria, glucosuria) and metabolic analysis during screening, follow-up 1 and follow-up 2
- Stool for helminths egg counts, including schistosome infections, *Strongyloides* larvae and protozoa during screening, enrolment, follow-up 1 and follow-up 2
- Blood prick test from volunteer’s finger for diagnosis of *Mansonella*, *Loa loa* and other filarial infections and blood glucose measurements during screening, follow-up 1 and follow-up 2
- Venous blood will be used to assess white blood cell counts, differential cell blood count, malaria parasites and fasting blood glucose levels, metabolic and immunological parameters during enrolment, follow-up 1 and follow-up 2

- HbA1c, cholesterol, triglycerides, HDL-C, LDL-C, AST, ALT, ALP, CGT, adipokines, Glucagon, Insulin, C-peptide, Ghrelin, GIP, GLP-1, HOMA-IR, serum creatinine, urea and urine albumin, LBP, EndoCAb, LPS, iFABP, α-2M, CRP, haptoglobin, SAA during screening (before treatment), follow-up 1 and follow-up 2
- Height, weight, waist circumference, body fat (using a weight scale body fat analyser), blood pressure, electrocardiogram measurements during screening, follow-up 1 and follow-up 2

9 SAMPLE COLLECTION AND DATA MONITORING

9.1 Study monitoring

Principal Investigators will monitor all aspects of the study, with respect to current GCP, to ensure compliance with government regulations. The objectives of a monitoring visit will be to ensure

i) Availability of the signed ICF (Annex ICF)
ii) GCP adherence to the protocol
iii) Accuracy and completeness of the CRFs (Annex CRF) with source data. During the monitoring visit, the investigator (and/or designee) and other study personnel should be available to discuss the study and any relating issues. Furthermore, all official study documentation must be available for review at all times.

9.2 Sample collection: skin biopsies and blood taking

Two skin biopsies will be taken during enrolment, follow-up 1 and follow-up 2 from the right and the left posterior iliac crest to determine the microfilaria load per milligram of skin. After disinfection of the skin areas, the skin will be snipped using a surgical device (2mm Holth Corneoscleral or Walzer punch). The wound will be covered with a plaster to avoid infection. Biopsies will be incubated in saline overnight and the microfilariae counted using a microscope. The skin biopsies will be weighed to determine the microfilariae per mg of tissue.

At baseline and both follow-up time points, after overnight fasting, volunteers will be also asked to provide urine (collected in clean pots labeled with the barcodes of each study participant) and venous blood samples (2x9ml in EDTA) will be drawn by venous puncture according to standard procedures for immune profiling (immunoglobulin subclasses, cytokines/chemokines via ELISA, PCR, multiplex ELISA, and immune cell profiling, leucocyte activation and cytokine production by flow cytometry) and metabolic/biochemical profiling (HbA1c, cholesterol, triglycerides, HDL-C, LDL-C, AST, ALT, adipokines, Glucagon, Insulin, C-peptide, Ghrelin, GIP, GLP-1, HOMA-IR). White blood cell counts, malaria parasites, as well as fasting blood glucose levels will be also determined in venous blood samples. One finger of the volunteer's hand will be pricked with a sterile lancet and 10µl of blood will be directly used to determine blood glucose levels and 20 µl of the same blood draw will be used to check for the presence of microfilariae. 50 µl of blood will be additionally transferred from the pricked finger into tubes containing 950 µl of 3% acetic acid and mixed thoroughly to lyse the red cells to confirm the results. The finger wound will be covered with a plaster to avoid infection. The risk, taking part in this study, is associated with the risk of developing a bruise at the point of needle insertion with possible colouration of the skin.

9.3 Intervention pilot study

An intervention study analyzing the kinetic of changes in the metabolic and immunological profiles after anti-filarial treatment will be conducted. All patients of the *M. perstans* infected obese and lean subjects will receive anti-filarial therapy (Fig. 1). The known treatment option for *M. perstans* infections with 200mg of doxycycline daily for 6 weeks will be used (28, 29) since ivermectin is known not to deplete the *Mansonella* adult worms and to have only modest effect on microfilariae (30). This doxycycline therapy will permanently sterilize the female adult *Mansonella* worms and lead to the death of the adult worms over time. All onchocerciasis patients will also receive the anti-filarial therapy with 200mg of doxycycline daily for 6 weeks, which was previously shown to sterilize the female adult *Onchocerca* worms and lead to the death of the adult worms over time (31, 32). Patients co-infected with *Onchocerca* and *Mansonella* will also be treated with 200mg of doxycycline daily for 6 weeks. As *L. loa* does not contain *Wolbachia* endosymbionts, doxycycline treatment will not affect *L. loa* co-infection. Onchocerciasis patients, *M. perstans* infected patients, *L. loa* infected individuals and endemic controls will receive single dose of 400 mg albendazole every three months with a total of 4 treatments for the elimination of soil transmitted helminths. Treatment of *L. loa* infected individuals with albendazole does not kill the adult worms, but reduces the microfilariae load in a safe and well tolerated manner. Treatment of onchocerciasis patients with ivermectin as originally intended is not an option, as the study site selected for our study received more than 10 years of ivermectin MDA and suboptimal responses were observed with reoccurring microfilaridermia within less than one month. As a 6-week treatment with doxycycline is not ethically acceptable for endemic controls, those participants will only receive albendazole treatments. Although *Mansonella* infections do not cause pathology and there is no MDA program for mansonellosis we are now treating all *Mansonella* patients with doxycycline. The benefit of the *Mansonella* patients to receive doxycycline may be a lower risk to acquire co-infections such as tuberculosis and HIV. On the other hand we hypothesize that clearance of the *Mansonella* infection by doxycycline therapy may have a negative effect on insulin sensitivity in obese patients, but this needs scientific evidence and was not sufficient to convince the participants of the study not to be treated for the duration of this study. An overview of the groups and subject numbers is provided in Fig.1.

Doxycycline (tablets, film-coated) will be administered ad personam by the study clinician directly in the villages under supervision (directly observed treatment, DOT) for 6 weeks. Doxycycline 200mg (2 tablets a 100mg) will be administered orally once daily for 6 weeks. The dosage will be reduced to 1 tablet a 100mg in participants with a body weight $\geq$ 40kg and < 50kg. Individuals below 40kg will not be treated with doxycycline.

All participants of this study will be also treated by the study clinician directly in the villages under supervision (directly observed treatment, DOT) with 400mg albendazole (one tablet) once every three months for a total of four treatments. This treatment clears intestinal helminths and is used for MDA treatment.

Participants positive for *L. Loa* will receive the above described albendazole treatment and will be followed up on after 12 and 18 months.

All subjects will be followed up at 12 and 18 months after treatment and sample collections as described under 6.2 will be performed. Patients will be screened for helminth infections and the impact of possible re-infections following doxycycline or ivermectin treatment will be taken into account. Compliance to the treatment will be confirmed by collecting empty drug containers during the visit of the study subjects. Subjects will be monitored throughout the study for any adverse effects.

Each treatment will be documented and potential severe adverse events will be documented in the CRF (see Annex CRF).

9.3.1 Side effects of doxycycline:

Very frequent (> 10%) side effects:
Infections and Infestations: Common cold (22%), influenza symptoms (11%)
Gastrointestinal: Nausea (up to 13.4%)
Nervous system: Headache (up to 26%)

Frequent (1-10%) side effects:
Metabolic and Nutritional: Increased blood lactate dehydrogenase (2%), increased blood glucose (1%)
Respiratory: Nasopharyngitis (5%), sore throat (5%), sinus congestion (5%), coughing (4%), sinus headache (up to 4%), sinusitis (3%), bronchitis (3%), nasal congestion (2%), pharyngolaryngeal pain (1%)
Infections and Infestations: infection (2%), fungal infection (2%), influenza (2%)
Reproductive Disorders: Menstrual cramp (4%), bacterial vaginitis (3.3%), vulvovaginal mycotic infection (2%)
Hepatic and Biliary: Increased aspartate aminotransferase (2%)
Body as a whole – general: Injury (5%), pain (up to 4%), back pain (up to 3%), back ache (2%)
Dermatologic: Rash (4%)
Gastrointestinal: Vomiting (8.1%), toothache (7%), tooth disorder (6%), dyspepsia (6%), diarrhea (up to 6%), periodontal abscess (4%), acid indigestion (4%), upper abdominal pain (2%), abdominal distention (1%), abdominal pain (1%), stomach discomfort (1%), dry mouth (1%)
Musculoskeletal: Joint pain (6%), muscle pain (1%)
Cardiovascular: Hypertension (3%), increased blood pressure (2%)
Psychiatric: Anxiety (2%)

Occasionally (0.1 to < 1%) side effects:
Gastrointestinal: Gum pain

Rare (< 0.1%) side effects:
Gastrointestinal: Adult tooth staining (at least 1 case)

Frequency not reported:
Hematologic: Hemolytic anemia, thrombocytopenia, neutropenia, eosinophilia
Metabolic and Nutritional: Hypoglycemia, anorexia
Infections and Infestations: Possible overgrowth of nonsusceptible organisms (superinfection)
Renal and Urinary: Dose-related rise in BUN
Reproductive Disorders: Vaginal itch, vaginal candidiasis
Hepatic and Biliary: Acute hepatocellular injury, cholestatic reactions, hepatotoxicity
Body as a whole – general: Microscopic brown-black discoloration of the thyroid gland
Dermatologic: Nail discoloration, phototoxicity, photoallergic reaction, photo-onycholysis, photosensitivity, maculopapular and erythematous rashes, erythema multiforme, Stevens-Johnson syndrome, toxic epidermal necrolysis, exfoliative dermatitis, hyperpigmentation
Gastrointestinal: Clostridium difficile associated diarrhea, esophageal irritation, ulceration, epigastric burning, black hairy tongue, Esophagitis and esophageal ulcerations (most took medication immediately before going to bed)
Nervous system: Sinus headache, dizziness, drowsiness, amnesia, paresthesias of body areas exposed to sunlight, phrenic nerve paralysis after sclerotherapy, benign intracranial hypertension resulting in significant loss of vision

Ocular: Diplopia, papilledema, loss of vision (associated with doxycycline-induced benign intracranial hypertension)
Hypersensitivity: Hypersensitivity reactions (including urticaria, angioneurotic edema, anaphylaxis, anaphylactoid purpura, serum sickness, pericarditis, exacerbation of systemic lupus erythematosus, drug rash with eosinophilia and systemic symptoms)
Immunologic: Autoimmune syndromes

Postmarketing reports:
Nervous system: Pseudotumor cerebri (benign intracranial hypertension), headache

9.3.2 Precautions to mitigate drug specific risks (Doxycycline)

Photosensitivity
Photosensitivity manifested by an exaggerated sunburn reaction has been observed in some individuals taking tetracyclines. Patients apt to be exposed to direct sunlight or ultraviolet light will be advised that this reaction can occur with tetracycline drugs. Treatment will be immediately stopped at the first evidence of skin erythema.

Growth and Development
All tetracyclines form a stable calcium complex in any bone-forming tissue. A decrease in fibula growth rate has been observed in prematures given oral tetracycline in doses of 25 mg/kg every six hours. This reaction was shown to be reversible when the drug was discontinued. Results of animal studies indicate that tetracyclines cross the placenta, are found in fetal tissues, and can have toxic effects on the developing fetus (often related to retardation of skeletal development). Evidence of embryotoxicity also has been noted in animals treated early in pregnancy. If any tetracycline is used during pregnancy or if the patient becomes pregnant while taking these drugs, the patient should be apprised of the potential hazard to the fetus. Therefore women taking part in the clinical trial will be informed in detail about the risk associated with a pregnancy during drug intake. Pregnancy tests will be carried out every week and if a positive pregnancy test occurs against all precautions, treatment will be stopped immediately.

Clostridium difficile –associated diarrhea
Clostridium difficile-associated diarrhea (CDAD) has been reported with the use of nearly all systemic antibacterial agents, including Moxifloxacin, with severity ranging from mild diarrhea to fatal colitis. Therefore participants in this study with diarrhea will be monitored especially for CDAD and in case of suspected or confirmed CDAD treatment will be immediately stopped and the patient will be treated for CDAD as appropriate (fluid and electrolyte management, protein supplementation, antibacterial treatment of *C. difficile*, surgical evaluation if clinically indicated) until recovery. Since CDAD has been reported to occur over two months after the administration of antibacterial agents, patients will be strongly encouraged to report any severe diarrhea to the research team also after completion of the treatment.

Superinfection
As with other antibiotic preparations, use of this drug may result in overgrowth of non-susceptible organisms, including fungi. If superinfection occurs, treatment will be immediately stopped and appropriate therapy instituted until recovery.

Benign Intracranial Hypertension (pseudotumor cerebri)
Bulging fontanels in infants and benign intracranial hypertension in adults have been reported in individuals receiving tetracyclines. These conditions disappeared when the drug was discontinued. Therefore treatment of

patients with a suspected benign intracranial hypertension (e.g. headache associated with gradual visual field defects, nausea, vomiting, drowsiness) will be stopped immediately and appropriate therapy will be given until full recovery.

Pregnancy and breastfeeding:
To avoid any complications, pregnant or breastfeeding women will be excluded from the clinical trial. Pregnancy tests will be carried out during the screening and right before the first treatment. Additionally, pregnancy tests will be repeated at follow-ups. In case of pregnancy in any group, treatment will be stopped immediately. All women will be informed in detail about the risks of getting pregnant during the informed consent procedure and their obligation and responsibility to use effective contraceptive methods excluding daily hormonal contraception as the trial drugs can reduce their efficacy.

Patients with creatinin, AST, ALT, and µ-GT levels exceeding two times the upper limit will not be treated with doxycycline. These values will be determined less than one month before doxycycline treatment.

9.3.3 Side effects of Albendazole

Very frequent (> 10%) side effects:
Hepatic: Elevated liver enzymes (up to 16%, mostly mild to moderate)
Nervous system: Headache (up to 11%)

Frequent (1-10%) side effects:
Dermtologic: Reversible alopecia (thinning of hair, moderate hair loss)
Gastrointestinal: Epigastric / abdominal pain, nausea, vomiting, upper gastrointestinal (GI) symptoms, GI disturbances
Hematologic: Leukopenia
Nervous system: Raised intracranial pressure, dizziness, neurological events
Other: fever, hyperpyrexia

Occasionally (0.1 to < 1%) side effects:
Dermtologic: itchiness, skin rashes
Gastrointestinal: diarrhea
Hepatic: hepatitis
Hypersensitivity: hypersensitivity reactions (including rash, pruritus, urticaria)
Nervous system: vertigo, meningeal signs

Rare (< 0.1%) side effects:
Gastrointestinal: pancreatitis
Genitourinary: proteinuria
Hematologic: low red cell count, pancytopenia, thrombocytopenia
Musculoskeletal: bone pain
Ocular: ocular maculopathy

Frequency not reported:
Hematologic: granulocytopenia, agranulocytosis
Hepatic: hepatotoxicity, hepatic abnormalities*, jaundice*, hepatocellular damage*
*severe hepatic adverse events reported during prolonged higher dose treatment
Ocular: retinal damage

Postmarketing reports:
Dermtologic: erythema multiforme, Stevens-Johnson syndrome, reversible alopecia (thinning of hair and moderate hair loss)
Gastrointestinal: diarrhea
Hematologic: aplastic anemia, bone marrow suppression (mainly in patients with liver disease), neutropenia, agranulocytosis, pancytopenia
Hepatic: acute liver failure, elevated liver enzymes and hepatitis
Musculoskeletal: rhabdomyolysis
Nervous system: somnolence, convulsion, headache
Ocular: blurred vision
Renal: acute renal failure
Other: asthenia, fever

9.3.5 Adverse events

Documenting, Recording and Reporting Adverse Events
At each contact with the subject, information regarding adverse events will be elicited by appropriate questioning and examinations and will be:

- immediately documented in the subject's medical record/source document
- The study clinicians/physicians will visit the participants in their villages every day during the treatment period to closely supervise possible (severe) adverse events. Additionally the phone numbers of the trial clinicians will be handed to the village health workers as well as to the participants. In case of an emergency between the visits, the clinicians have to be called and will come to the participants whenever needed. In case the participant has to go to a hospital for further examinations and treatment, the transport and medical care will be paid by the research team. An insurance for participants and the medical personal involved in the project and directly in charge of the patients will be provided.

9.4 Assessment of immunological parameters

Plasma will be aliquoted (2x2ml) in cryotubes, immediately frozen and stored for determination of the immunological parameters. Whole blood cells will be stained for distinct immune populations and measured by flow cytometry. Whole blood cells will also be used directly in cell culture assays to determine intracellular cytokine profiles upon stimulation. A fraction of whole blood cells will be stored at -80°C to repeat the assays or verify another parameter which is requested by reviewers during publication.

9.5 Source documentation

Complete source documentation (e.g. laboratory test reports, medical records) will be required for each participant for the entire duration of the study. Case Report Forms (Annex CRF) will be used to record data for subjects enrolled in the study. The investigator is responsible for the accuracy and completeness of the data reported to the study sponsor. Data reported in the CRFs derived from source documents should be consistent with them or the discrepancies should be explained.

9.6 Study documentation, data safety and confidentiality

Pseudonymity of the study participants will be maintained by assigning study participants a unique study identification number. All data, blood samples and laboratory results will be recorded and analyzed with this unique identification number and no personal identifiers. All information collected, including names, specific addresses, etc. that could identify participating subjects will be kept confidential and available only to the investigators and authorized study personnel.

All data will be double entered by trained personnel directly in Cameroon and subsequently cleaned by the Data Manager in Bonn using the data management program REDCap®. The data bases will be password protected and access to password will be authorized by the Principal Investigators. Electronic data files will be stored on operated and dedicated servers in the respective study laboratories. Analyses of the data will be performed on copies of the original data files ensuring raw data accessibility at all times. Regular backups to an external hard drive will be carried out ensuring protection against data loss. The anticipated funding body (DFG) hold an annual meeting to check the progress and status of each project.

9.7 Data Collection & Management

All necessary precautions are taken to ensure that our research data is stored, archived and disposed of in a safe and secure manner during and after the conclusion of the research project. Laboratory notes are reported daily in the researcher's personal laboratory book. These books are the property of the Institute and remain there even after the departure of the scientist. Regular electronic backups are made of the raw data from computers attached to institute equipment such as the flow cytometer. Data for publications are collected and stored on the Institute's server which is protected from external tampering by the usual precautions.

The investigator has ultimate responsibility for the accuracy, authenticity, timely collection and reporting of all clinical, safety, laboratory data entered on the Case Report Forms (CRFs). Data will be captured using paper CRF specially designed for the study and approved by the IRBs. The CRFs must be signed by the investigator or by an authorized staff member to attest that the data contained on the CRFs is true. Any corrections to entries made in the CRFs, source documents must be dated, signed and explained (if necessary) and should not obscure the original entry. All paper CRFs will be secured in fireproof locking cabinet. All study personnel participating will undergo training in the procedures to be used in the study.

Research data collected on paper CRFs will be transcribed and entered onto the REDCap (Research Electronic Data Capture: http://project-redcap.org/) system using double data entry. The EDC application is a secure encrypted web application for building and managing online surveys and databases specifically geared to support data capture for research studies. REDCap meets regulatory requirements for GCP/GCLP, 21 CFR Part 21 and HIPAA compliant with full audit trails capability for tracking data manipulation and user activity, as well as automated export procedures for seamless data downloads to Excel, PDF, and common statistical packages (SPSS, SAS, Stata, R). The equipment for data transfer may include laptops, or mobile applications for tablet computers and/or smartphones with either Android or iOS operating systems. All electronic tools will be password protected.

Study data will be managed using REDCap electronic data capture tools hosted at the University Hospital Bonn. REDCap (Research Electronic Data Capture) is a secure, web-based application designed to support data capture for research studies, providing: 1) an intuitive interface for data entry; 2) audit trails for tracking

data manipulation and export procedures; 3) automated export procedures for seamless data downloads to common statistical packages; and 4) procedures for importing data from external sources. Details on data management (procedures, responsibilities, data corrections, if any, which may be made by Data Management staff themselves, etc.) will be described in a data management plan prior to the trial. During the trial, the performance of data management and any deviations from the data management plan will be documented in a data management report. Before any data entry is performed, the trial database will be validated and the technical specifications of the database will be documented in a variable plan. Processing of data is done via Double-Data-Entry. The two entries will then be compared with each other and verified. An audit trail will be created to provide an electronic record of which data were entered or subsequently changed, by whom and when. SAS software will be used to review the data for completeness, consistency and plausibility. The checks to be programmed will be specified beforehand in a data validation plan, as required by the subject protocol. After running the check programs, the resulting queries will be sent to the investigator for review of his/her data. Answered queries will also be entered twice, verified and the updated data will then be transferred to the database. All programs which can be used to influence the data or the data quality will be validated (e.g. check programs, programs used for the input of external data, etc.).

All data will be checked for consistency and plausibility by the monitor and by the data management. Inconsistencies will be queried and discussed with the investigator. After data clearance the data base will be locked and data will be used for statistical analysis.

9.8 Retention of records

All written forms (i.e. consent and data collection forms) will be stored in the study laboratories in Cameroon and Bonn. All forms will be labelled and filed in lockable filing cabinets with the Study Protocol Name, Principal Investigators' names and collection dates. Keys will be kept with the principal investigators or the persons responsible for data management. Study-related documents will be maintained by the investigator for a period of 15 years following completion of study. The electronic database will be stored for 15 years by the Principal Investigators. All data will be readily available for review upon request by the national authorities and the IRBs.

9.9 Protocol revisions

Revisions in the study protocol can only be performed following renewed permission from the local ethics authorities. This does not apply to changes made to reduce discomfort or avert risk to study participants.

9.10 Sample transport from Cameroon to Bonn

Analysis from blood and urine samples will include the glycemic and biochemical analyses via the Reflotron system and multiplex ELISA measurements as well as multicolor flow cytometric analyses of intracellular cytokine producing T cells from blood samples will take place in Bonn, Germany. We do not intend to use the obtained material for commercial purposes. Therefore the project represents an ABS constellation (Tabelle in Punkt 3. DFG-Vordruck 1.021 – 5/08) that requires for export of biological material (blood and urine samples) to the German partner institute a simple ABS agreement (Tabelle in Punkt 3. DFG-Vordruck 1.021 – 5/08) and a standardized Material Transfer Agreement will be used ensuring and promoting technology transfer and long-term sharing of the benefits derived from those samples

9.11 Termination of the trial for individual subjects

9.11.1 Termination by the participant

A patient may decide to withdraw from the study at any time and for any reason. The Investigator should attempt to determine the reason for the subject’s decision. There will be no disadvantage for the participant as a result of a withdrawal. If a participant does not return for a scheduled visit, every effort should be made to contact the participant. The investigator should inquire about the reason for withdrawal and the participant should be followed-up regarding any unresolved adverse events, if possible. In any circumstance, every effort should be made to document the participants’ outcome. Therefore all participants, even if the participant was withdrawn from the trial treatment, will be encouraged to come for the follow-up visits.

9.11.2 Termination by the investigator

The investigator may withdraw a patient for any of the following reasons:

- An individual subject’s decision
- Any clinical adverse events, laboratory abnormality or other medical condition or situation such that continued participation in the study would not be in the best interest of the subject. Subjects will be followed for the duration of the study for indicated safety assessments.
- Non-compliance with study procedures to the extent that it is potentially harmful to the subject or to the integrity of the study data.
- A change in the subject’s baseline condition after enrolment so that the subject no longer meets the inclusion/exclusion criteria.

In any circumstance, every effort should be made to document the participants’ outcome. Therefore all participants, even if the participant was withdrawn from the trial treatment, will be encouraged to come for the follow-up visits.

9.12 Study Monitoring

To ensure accurate, complete, consistent, and reliable data, and ensure the patients´ safety, the investigator’s site and trial procedures will be monitored by the ministry of public health, the Division of Operational Research in Health (DROSS). The public health’s representative will visit the site:

- to evaluate the progress and recruitment of the trial,
- to review the source documents and CRFs for protocol compliance, accuracy and validation,
- to assess facilities and equipment,
- to check for protocol compliance,
- to assure the (severe) adverse events reporting,
- to verify proper handling and dispensing of the IMP(s), and other factors.

The investigator agrees to cooperate with the monitor to ensure that any problems detected in the course of these monitoring visits are addressed and resolved, and therefore ensures the accuracy and consistency of the trial with GCP and all applicable laws. The investigator allows the monitor to have access to all trial related original data and documents relevant for the monitoring of the trial.

10 STATISTICAL CONSIDERATIONS

10.1 Sample size justification and statistical analysis

This is a partially randomized controlled open label pilot trial to gain first experience on the impact of infections with the filarial nematodes *O. volvulus* and *M. perstans* on the metabolic profile and the systemic immune response in lean and obese patients. As there is limited data that exists to inform on study design, we will use 400 persons per group (*O. volvulus* infected, *M. perstans* infected, and endemic non-helminth-infected persons) with half of the participants of each group having a BMI of ≥30 and the other half of below 25. Thus, cases (those with active worm infections) and controls (worm infection free) will be included at a case: control ratio of 1:1:1. All *M. perstans* infected patients and all *O. volvulus* infected persons will further be treated in an interventional pilot study to test the changes in the metabolic profile and the systemic immune responses following treatment with doxycycline.

Our primary endpoint will be quantitative changes in insulin resistance (HOMA-IR). The logarithmic transformed HOMA-IR values will be compared between the three obese subgroups with a two-step test procedure. In the first step a one factorial analysis of variance will be used to test the hypotheses of equal mean log(HOMA-IR) values in the three groups at a level of 5%. In case that this hypothesis can be rejected the mean log(HOMA-IR) values will be checked for equality for each pair of groups with a two-sided t-test at a level of 5% for each test. This test procedure maintains a multiple level of 5%. From literature data (33) we expect the logarithm of the HOMA-IR to be normally distributed with a standard deviation of 0.83. With the planned sample size of 200 cases per group the intended test procedure then has a power of 80% to detect a deviation of 0.3 in the mean log(HOMA-IR) values between two of these groups. This is equivalent to reduction of 25% of the HOMA-IR in comparison between these two groups.

11 DELIVERABLES

The objectives outlined within this proposal are a work package within a proposal that was submitted to the DFG by mid of October 2017. The application was written in response to the call for German-Africa cooperation projects in infectiology and was invited for submission of the full proposal after which it has been approved for funding. The requested funding is for three years and we anticipate that the duration of the whole project will be 8 years.

12 RESULTS

Anticipated outcome:

This project will yield a better understanding into how filarial nematode infections influence systemic immune responses and impact the glycemic and metabolic profiles. We expect that infections with *M. perstans* and *O. volvulus* lead to reduced obesity-induced systemic inflammatory immune responses and correlate with lower glucose, HbA1c, cholesterol and LDL-C levels, improved liver function and pancreatic and gut hormone release as well as improved insulin sensitivity. Results of this study will help us to assess the impact of anti-filarial treatment programs on the development of metabolic diseases like T2D.

13 HUMAN RIGHTS PROTECTION

13.1 Research Ethics Committee

The Principal investigators will be responsible for obtaining ethical approval from all Ethic Committees relevant to this study (listed in 3.5). Before the start of the study, appropriate documents (including Study Protocol, Informed Consent Form (from study participants and from parents in case of study participants less than 21 years) and Patient Information Sheet, (Annex ICF)) will be submitted for ethical approval. A copy of the study approval (including a copy of all annexes) will be maintained in the study document binder and a copy will be supplied to the sponsor. The investigator will document study progress, in the form of an annual report, which will be submitted to the DFG.

13.2 Informed Consent Forms (ICF)

Regulatory requirement necessitates obtaining and documenting informed consent (from study participants and from parents in case of study participants less than 21 years), GCP and ethical principles along the Declaration of Helsinki 1964 (amended, most recently in 2004). All consent forms must accompany the study protocol and submitted for approval by the relevant ethic committees.

13.3 Benefits

The benefits of the volunteers´ participation will be a free treatment against accompanying infections that might be diagnosed during recruitment (malaria, schistosomiasis and gastrointestinal parasites) with the relevant standard therapy. Furthermore, all patients infected with *O. volvulus* and *M. perstans* will be treated with the only filaricidal treatment available, doxycycline, and all participants of this study (including endemic controls) will receive albendazole treatment, which eliminates intestinal helminths and is regularly used for MDA. In *L. loa* patients albendazole treatment leads to a safe and well-tolerated decline of microfilariae. Within this project newly diagnosed diabetes patients will be referred to a local diabetologist. ECG results will be told to subjects and subjects with abnormal results will be referred to a local cardiologist. The patients will be further informed about their glycemic status, lipid profiles, liver and kidney function following each visit.

13.4 Risks

During the study period blood sampling and skin snipping will be carried out as invasive intervention (a total of 6 blood draws in 12-month intervals is envisioned), which may bear a potential risk. In general those treatments are well tolerated. However, despite appropriate procedure, hematomas may develop that disappear after a few days. Dizziness, indisposition, or secondary bleedings may occur at the injection/snipping site. In really rare cases small blood clots or inflammatory reactions at the injection/snipping site may occur. Really rare cases further include injuries of the arteries or nerves, which could be irreversible in individual cases and cause chronic pain. In order to prevent any infection during blood sampling and skin snipping, high hygienic standards will be applied. The skin will be disinfected before blood draw/skin snipping and sterile devices will be used for sampling. The wound will be subsequently covered with a plaster to avoid infection. Every unexpected event in context with the blood sampling and skin snipping will be treated by the trial clinician until complete resolution.

Furthermore, side effects of doxycycline and albendazole therapy may occur as described under 6.3.1 and 6.3.3 and will be mitigated as described under 6.3.2.

13.5 Confidentiality

All study-related information will be securely stored at the study site. All participant information will be stored in locked filling cabinets with limited access. Laboratory specimens, reports, study data collection, process and administrative forms will be identified by ID number to maintain participant confidentiality. Computer data entry will also be carried out using ID numbers. Personal information such as patient names, hospital numbers and addresses will not be recorded on the CRFs used. Other documents that link participant ID numbers to identifying information will be stored in separate locked files and all computer data, data entry programmes and networking programs will be password protected and subject to limited access. If records are required for examination, this information will be blanked out; however, a secure record of the linkages between individuals and their records will be maintained. Clinical information will not be released without written permission of the subject, except as necessary for monitoring by IRB, the national regulatory authority, or the sponsor's designee. All such information will be anonymized.

13.6 Compensation & patient insurance

Participants will not receive any monetary compensation.

Every subject participating in the trial will be insured against any trial-related illness/injuries pursuant to the legal requirements which may occur during the trial. Excluded from this, however, are injuries to health and deterioration of illnesses already in existence which would have continued to exist even if the subject had not taken part in the clinical trial. However, newly diagnosed diabetes patients will not be treated for the duration of the project. The investigator will inform the subject of the existence of the insurance, including the obligations arising from it. The participants must be afford access to insurance documents and provided with a copy of the general conditions of insurance on request. The insurance cover is jeopardized if the subject fails to immediately report (within 7 days) to the investigator or responsible physician any injury to health which might have resulted from the participation in the clinical trial, or if she/he undergoes any other medical treatment (except for emergency treatment) without the investigator's knowledge before her/his participation in the clinical trial has officially ended. In case of any health impairment the subject is obliged to notify the investigator as soon as possible. The investigator is then obliged to notify the insurance and additionally to make a report to the sponsor. The subject insurance will be arranged by the sponsor delegated person.

13.7 Study Registration

The study will be registered at ISRCTN after approval was obtained and before the enrolment of participants.

13.8 Data safety and monitoring committee (DMSC)

A single central DSMC will be established with a charter that defines the roles and responsibilities and details of meeting frequency and communications. The DSMC will, in addition to the Authorities/IRBs involved, have a chance to comment on the content of the protocol. The Division of Operational Research in Health (DROSS) by the ministry of public health will act as DMSC.

14 LIST OF ABBREVIATIONS

CRF	Case Report Form
GCP	Good Clinical Practices
ICF	Informed Consent Form

IMMIP	Institute for Medical Microbiology, Immunology and Parasitology
IVM	Ivermectin
MDA	Mass Drug Administration
Mf	Microfilariae
SOP	Standard Operating Procedures
T2D	Type 2 Diabetes

15 REFERENCES

1. Aravindhan V, *et al.* (2010) Decreased prevalence of lymphatic filariasis among diabetic subjects associated with a diminished pro-inflammatory cytokine response (CURES 83). *PLoS Negl Trop Dis* 4(6):e707.
2. Chen Y, *et al.* (2013) Association of previous schistosome infection with diabetes and metabolic syndrome: a cross-sectional study in rural China. *J Clin Endocrinol Metab* 98(2):E283-287.
3. Hays R, Esterman A, Giacomin P, Loukas A, & McDermott R (2015) Does Strongyloides stercoralis infection protect against type 2 diabetes in humans? Evidence from Australian Aboriginal adults. *Diabetes Res Clin Pract* 107(3):355-361.
4. Wiria AE, *et al.* (2015) Infection with Soil-Transmitted Helminths Is Associated with Increased Insulin Sensitivity. *PLoS ONE* 10(6):e0127746.
5. Hussaarts L, *et al.* (2015) Chronic helminth infection and helminth-derived egg antigens promote adipose tissue M2 macrophages and improve insulin sensitivity in obese mice. *FASEB journal : official publication of the Federation of American Societies for Experimental Biology* 29(7):3027-3039.
6. Berbudi A, *et al.* (2016) Filarial Infection or Antigen Administration Improves Glucose Tolerance in Diet-Induced Obese Mice. *Journal of innate immunity* 8(6):601-616.
7. Wu D, *et al.* (2011) Eosinophils sustain adipose alternatively activated macrophages associated with glucose homeostasis. *Science* 332(6026):243-247.
8. Yang Z, *et al.* (2013) Parasitic nematode-induced modulation of body weight and associated metabolic dysfunction in mouse models of obesity. *Infect Immun* 81(6):1905-1914.
9. Bhargava P, *et al.* (2012) Immunomodulatory glycan LNFPIII alleviates hepatosteatosis and insulin resistance through direct and indirect control of metabolic pathways. *Nature medicine* 18(11):1665-1672.
10. Hams E, *et al.* (2016) The helminth T2 RNase omega1 promotes metabolic homeostasis in an IL-33- and group 2 innate lymphoid cell-dependent mechanism. *FASEB journal : official publication of the Federation of American Societies for Experimental Biology* 30(2):824-835.
11. Tahapary DL, *et al.* (2017) Effect of Anthelmintic Treatment on Insulin Resistance: A Cluster-Randomized Placebo-Controlled Trial in Indonesia. *Clinical infectious diseases : an official publication of the Infectious Diseases Society of America*.
12. International Diabetes Federation. IDF Diabetes Atlas eB, Belgium:International Diabetes Federation (2015).
13. Surendar J, Indulekha K, Hoerauf A, & Hübner MP (2016) Immunomodulation by helminths: similar impact on type 1 and type 2 diabetes? *Parasite Immunol*.
14. Berbudi A, Ajendra J, Wardani AP, Hoerauf A, & Hübner MP (2016) Parasitic helminths and their beneficial impact on type 1 and type 2 diabetes. *Diabetes Metab Res Rev* 32(3):238-250.
15. Ajendra J, Berbudi A, Hoerauf A, & Hübner MP (2016) Combination of worm antigen and proinsulin prevents type 1 diabetes in NOD mice after the onset of insulitis. *Clin Immunol* 164:119-122.
16. Hübner MP, Stocker JT, & Mitre E (2009) Inhibition of type 1 diabetes in filaria-infected non-obese diabetic mice is associated with a T helper type 2 shift and induction of FoxP3+ regulatory T cells. *Immunology* 127(4):512-522.

17. Hübner MP, *et al.* (2012) Helminth Protection against Autoimmune Diabetes in Nonobese Diabetic Mice Is Independent of a Type 2 Immune Shift and Requires TGF-beta. *J Immunol* 188(2):559-568.
18. Larsen CM, *et al.* (2007) Interleukin-1-receptor antagonist in type 2 diabetes mellitus. *N Engl J Med* 356(15):1517-1526.
19. Stanley TL, *et al.* (2011) TNF-alpha antagonism with etanercept decreases glucose and increases the proportion of high molecular weight adiponectin in obese subjects with features of the metabolic syndrome. *The Journal of clinical endocrinology and metabolism* 96(1):E146-150.
20. Yuan M, *et al.* (2001) Reversal of obesity- and diet-induced insulin resistance with salicylates or targeted disruption of Ikkbeta. *Science (New York, N.Y.)* 293(5535):1673-1677.
21. Winer S, *et al.* (2009) Normalization of obesity-associated insulin resistance through immunotherapy. *Nature medicine* 15(8):921-929.
22. Nishimura S, *et al.* (2009) CD8+ effector T cells contribute to macrophage recruitment and adipose tissue inflammation in obesity. *Nat Med* 15(8):914-920.
23. Talukdar S, *et al.* (2012) Neutrophils mediate insulin resistance in mice fed a high-fat diet through secreted elastase. *Nat Med* 18(9):1407-1412.
24. Wensveen FM, *et al.* (2015) NK cells link obesity-induced adipose stress to inflammation and insulin resistance. *Nat Immunol* 16(4):376-385.
25. Olefsky JM & Glass CK (2010) Macrophages, inflammation, and insulin resistance. *Annu Rev Physiol* 72:219-246.
26. Chinetti-Gbaguidi G & Staels B (2011) Macrophage polarization in metabolic disorders: functions and regulation. *Current opinion in lipidology* 22(5):365-372.
27. Guigas B & Molofsky AB (2015) A worm of one's own: how helminths modulate host adipose tissue function and metabolism. *Trends Parasitol* 31(9):435-441.
28. Coulibaly YI, *et al.* (2009) A randomized trial of doxycycline for Mansonella perstans infection. *N Engl J Med* 361(15):1448-1458.
29. Hoerauf A (2009) Mansonella perstans--the importance of an endosymbiont. *N Engl J Med* 361(15):1502-1504.
30. Schulz-Key H, *et al.* (1993) Efficacy of ivermectin in the treatment of concomitant Mansonella perstans infections in onchocerciasis patients. *Trans R Soc Trop Med Hyg* 87(2):227-229.
31. Hoerauf A, *et al.* (2008) Wolbachia endobacteria depletion by doxycycline as antifilarial therapy has macrofilaricidal activity in onchocerciasis: a randomized placebo-controlled study. *Med Microbiol Immunol* 197(3):295-311.
32. Wanji S, *et al.* (2009) Community-directed delivery of doxycycline for the treatment of onchocerciasis in areas of co-endemicity with loiasis in Cameroon. *Parasit Vectors* 2(1):39.
33. Tahapary DL, *et al.* (2017) Effect of Anthelmintic Treatment on Insulin Resistance: A Cluster-Randomized, Placebo-Controlled Trial in Indonesia. *Clin Infect Dis* 65(5):764-771.
34. Omarine Nlinwe N, Larissa Kumenyuy Y, Precious Funwi C. Establishment of hematological reference values among healthy adults in Bamenda, North West region of Cameroon. Anemia. 2021;2021:6690926.
35. Oloume ME, Mouliom A, Melingui BF, Belinga S, Nana JS, Tejiokem M, et al. Haematological values in a healthy adult population in Yaoundé, Cameroon. Afr J Lab Med. 2019;8(1):852.
36. Kueviakoe I, Segbena A, Jouault H, Vovor A, Imbert M. Hematological reference values for healthy adults in Togo. ISRN Hematol. 2011;10:5. https://doi.org/10.5402/2011/736062
37. Troussard X, Vol S, Cornet E, et al. Étude des valeurs normales de l'hémogramme chez l'adulte: Un besoin pour une meilleure interprétation et pour l'accréditation du laboratoire. Ann Biol Clin 2014;72(5):61–81.
